# Unlocking the Quality Potential of Liberoid Coffee: Advances in Composition, Processing, and Microbial Fermentation

**DOI:** 10.1111/1541-4337.70503

**Published:** 2026-05-13

**Authors:** Noor Ariefandie Febrianto, Fan Zhu

**Affiliations:** ^1^ Indonesian Coffee and Cocoa Research Institute (ICCRI) PT. Riset Perkebunan Nusantara Jember Indonesia; ^2^ School of Chemical Sciences The University of Auckland Auckland New Zealand

**Keywords:** caffeine, Excelsa, Liberica, phenolic compound, post‐harvest processing, sensory quality, specialty coffee, starter culture, phenolic acid, sustainable crop

## Abstract

Liberoid coffee (*Coffea liberica* group), comprising varieties of Liberica and Excelsa coffee types, is gaining attention for its unique chemical composition and sensory potential. However, its quality remains under‐optimized due to limited understanding of genotype‐specific traits and processing responses. This review synthesizes recent findings on the proximate composition, bioactive compounds, and volatile profiles of Liberoid coffee, highlighting key differences from Arabica and Robusta. Emphasis is placed on the role of post‐harvest processing, particularly in vitro fermentation using defined microbial starters, in modulating flavor precursors, reducing alkaloids, and enhancing Maillard reaction products. Liberoid beans’ special chemical and physicochemical properties, coupled with their thick pulp and low bean yield, necessitate tailored processing strategies. Controlled fermentation offers a promising route to improve mouthfeel, reduce bitterness, and develop differentiated flavor profiles. Future research should prioritize microbial safety, fermentation optimization, and genotype‐specific processing to support the development of high‐quality Liberoid coffee with consistent sensory and functional attributes.

**Abbreviations**
AI: artificial intelligence
CGA: chlorogenic acid
CGAE: chlorogenic acid equivalent
CQA: caffeoylquinic acid
DB: dry basis
EPM: emerging processing method
GCB: green coffee bean
HS: horned skin
LAB: lactic acid bacteria
RB: roasted bean
RPM: rotation per minute
SCA: Specialty Coffee Association
TPC: total phenolic content
WPB: wet parchment bean

## 1 Introduction

Liberoid coffee is an emerging coffee variety that has started to gain importance in the coffee market (K. W. T. Lee 2024). “Liberoid” is a general term to refer to the group of *Coffea liberica*. This consists of Liberica coffee (from *C. liberica* var. *liberica*) and Excelsa coffee (from *C. liberica* var. *dewevrei*) (Davis et al. 2022, 2025). There has been increasing interest in Liberoid, mainly due to its high adaptability to marginal soil conditions (Wibisono et al. 2024), pest and disease resistance (Davis et al. 2022; Hafif et al. 2024), unique taste of its coffee beans (Purwanto et al. 2024), novel products (Izani et al. 2026), and its potential to complement both Robusta (*Coffea canephora* var. *robusta*) and Arabica (*Coffea arabica*) coffee, in terms of cultivation and bean quality (Davis et al. 2022; Saw et al. 2015). Its development is currently localized, particularly in Indonesia, the Philippines, Malaysia, Fiji, Vietnam, Ethiopia, India, and Uganda. Geographical condition was reported to affect the quality of coffee beans (Tieghi et al. 2024; Yusmaini and Fadhil 2025). Its interaction with genetics and post‐harvest treatment significantly shapes the chemical and sensorial properties of the coffee (Tieghi et al. 2024). However, these aspects of Liberoid coffee are not yet well understood.

Traditional and novel processing methods are being used to optimize the flavor of Liberoid coffee (de Melo Pereira et al. 2019; Febrianto and Zhu 2023). However, recent studies in these areas have yielded inconsistent results (Septiana et al. 2025; Salahuddin et al. 2025; Shah et al. 2025). This is mostly due to the lack of understanding of the mechanism of chemical changes during Liberoid coffee bean processing. Comprehensive information about the baseline chemical composition and the effect of post‐harvest processing on the chemical and sensorial properties of Liberoid coffee is needed. It is important to adjust the processing conditions suitable for Liberoid coffee beans to fully optimize the sensory potential.

Previous reports were mainly based on the studies of Arabica and Robusta coffee beans. They are mainly focused on chemical composition (Fabella‐Garcia et al. 2025); processing (Febrianto and Zhu 2023); biological properties (Machado et al. 2024); relation between chemical composition, processing, and physiological properties (Asadullah et al. 2025); traceability/supply chain (Ruggieri et al. 2026); in‐vivo/in‐vitro activity and availability of bioactives (Rojas‐González et al. 2022; Abidin et al. 2025; Pumaras et al. 2024); sensorial profiles (Soares et al. 2025); omics (Revelo‐Romo et al. 2025); analytical method (Urugo et al. 2025); artificial intelligence (AI)‐assisted analysis (Manansala and Paglinawan 2024); and by‐product utilization (Buyong and Nillian 2023; Wong and Nillian 2023). However, the studies of Liberoid coffee are mostly scattered. A review on the genetic variations in composition and processing including alternative methods of Liberoid coffee beans is not available yet. Comprehensive information about these topics could help to optimize the current processing methods of Liberoid coffee beans to obtain desired sensorial, functional, and nutritional properties of the beans and brew. This will support the rise of Liberoid coffee as a novel coffee variety.

## 2 Scope and Approach of the Review

This comprehensive review focuses on chemical constituents, particularly sensory‐related compounds (such as flavor precursors and volatiles), as well as bioactive compounds and sensory properties of Liberoid coffee beans as affected by their genetics and processing conditions. The implementation of novel in vitro fermentation technologies is also discussed.

The literature review primarily focused on studies published within the last 5 years; however, some older reports were included due to the limited availability of recent research on certain aspects of Liberoid coffee. Preference was given to papers published in English. As most relevant studies have been done in Southeast Asia, particularly in Indonesia, Malaysia, and the Philippines, locally published papers in regional languages of significant relevance were also considered. Data from previous research were systematically organized in chronological order to ensure clarity and coherence. Selected studies were discussed in greater depth to highlight similarities and discrepancies among research studies with comparable objectives.

This review aims to identify research gaps in the current understanding and development of Liberoid coffee. Findings from diverse studies, including those focusing on Arabica and Robusta, were critically compared and analyzed. Priority was given to studies providing comparative data with clearly defined sample identities in terms of origin, species, and genotype. Liberoid coffee is extensively cultivated in several regions of Asia; however, in certain areas such as Indonesia, misidentification between Liberica and Excelsa varieties remains common, leading to potential data inconsistencies. Accurate documentation of sample sourcing and identification procedures is therefore essential to ensure data reliability and minimize bias. In this review, the term “coffee beans,” unless otherwise specified, refers to Liberoid green coffee beans (GCBs). The term “Liberoid” encompasses all varieties related to *Coffea liberica*. Samples with confirmed identification are referred to by their respective species: “Liberica” denotes *Coffea liberica* var. *liberica*, while “Excelsa” denotes *Coffea liberica* var. *dewevrei*.

## 3 Liberoid Coffee Genetics, Production, and Distribution

The report on the genetic aspect and the distribution of Liberoid coffee is currently limited. *Coffea liberica* var*. dewevrei*, *C. liberica* var. *koto*, and *C. liberica* var. *liberica* were among wild coffee species/varieties found in Central African Republic, Cameroon, and Ivory Coast, respectively (Campa et al. 2004, 2005). The origin and distribution of Liberoid coffee in Asia and Africa before the 21st century were previously summarized (Davis et al. 2022). Liberoid coffee production currently existing is the remnant of pre‐Robusta commercialization. It could not compete with Robusta varieties in terms of coffee leaf rust resistance and with Arabica in terms of flavor (McCook 2019; Davis et al. 2022). Currently, Liberoid coffee represents a minor portion of the global coffee trade (less than 2%). However, it serves as a vital regional commodity in Southeast Asia, particularly on the peatlands of Indonesia (e.g., Meranti Islands, Jambi, and Bengkulu), where its production is expanding due to its unique environmental resilience (Waluyo and Nurlia 2017; Wibisono et al. 2024).

Liberoid coffee has suffered from a taxonomic confusion, mainly on Liberica and Excelsa. They share some similarities in the genetic expression of their agronomical features (Baltazar and Buot 2019). However, under the same cultivation conditions, the leaf architectural feature was found to be different between Liberica and Excelsa (Baltazar and Buot 2019). The differentiation can be based on the size of coffee beans. Coffee beans of Arabica, Robusta, Liberica, and Excelsa are different in appearance (Figure S1). Excelsa coffee beans exhibit a rounded morphology, with seed dimensions ranging from 5 to 12 mm, closely resembling those of Arabica; in contrast, Liberica beans are significantly larger and more elongated, with seed lengths spanning 10 to 18 mm, exceeding the size range observed in both Excelsa and Arabica cultivars (Davis et al. 2022). Nevertheless, further comparative studies involving diverse varieties and genotypes of Liberoid coffee are warranted, particularly with respect to plant morphology and bean architecture across varying geographical and agronomic conditions. Such investigations would facilitate accurate differentiation of Liberoid coffee by growers and help to mitigate ongoing confusion and misclassification within the species.

Genomic analysis revealed more diverse varieties of Liberoid coffee. The current Liberoid coffee classification includes another variety of Liberoid coffee, namely *C. liberica* var. *klainei* (Davis et al. 2025). This variety was previously misclassified as Liberica due to their high similarity in agronomic traits. As *C*. *liberica* var. *klainei* was reinstated as part of the Liberoid group, the distribution of Liberoid coffee in the African region has been well mapped. Central African countries, such as the Republic of the Congo, Cameroon, the Democratic Republic of Congo, the Central African Republic, South Sudan, and Uganda, are mainly dominated by Excelsa coffee. Liberica coffee is more dominant in upper West Africa (Sierra Leone, Liberia, Ivory Coast, Ghana, and Nigeria). *Coffea liberica* var. *klainei* is thus centered in West‐Central Africa, including Cameroon, Gabon, the Republic of Congo, and Angola (Davis et al. 2023, 2025). Considering that most Liberica plants in Asia were introduced from Africa, this reinstatement of *C. liberica* var. *klainei* as a different variety to that of Liberica coffee/*C*. *liberica*/*C*. *liberica* var. *liberica*, tracing back the origin of planted Liberica coffee in Asia, is needed to further confirm the genetic relationship. This information will be beneficial in the breeding program carried out for Liberoid coffee.

Efforts to identify coffee varieties and associate them with geographical origin have been reported, employing approaches beyond DNA‐based analysis. Among these, sensorial profiling has been explored as a differentiating tool (di Donfrancesco et al. 2019); however, its reliability is very limited due to the substantial influence of post‐harvest processing on sensory attributes (di Donfrancesco et al. 2019; Tieghi et al. 2024). In contrast, chemical composition offers a more objective basis for varietal discrimination, particularly between Arabica and Robusta coffee (Piotr Konieczka et al. 2020). Notably, caffeine content differs among species, with Liberoid beans containing 0.94%–1.24% dry basis (DB), markedly lower than Robusta (2.14% DB) and comparable to Arabica (1.19% DB) (Campa et al. 2005; Sualeh et al. 2020; Prakash et al. 2022). The chemical profiles of Arabica and Robusta beans have been extensively characterized, including amino acids (Dong et al. 2015; Kulapichitr et al. 2019), fatty acids (Mehari et al. 2019), organic acids (Peñuela‐Martínez et al. 2018), phenolic compounds (Badmos et al. 2019), and volatiles (Debona et al. 2025). However, data on Liberoid coffee remains scarce. Comprehensive chemical characterization of Liberoid beans, particularly in relation to sensory‐relevant compounds, is urgently needed to support optimized processing strategies and to unlock their potential as novel food ingredients.

## 4 Liberoid Coffee Bean Processing: Current Methods, Emerging Technologies And Factors Influencing Method Selection

### 4.1. Current Methods in Liberoid Coffee Bean Processing

The current processing of Liberoid coffee beans is done by imitating the current practices for Arabica/Robusta beans, namely wet, semi‐wet, and dry processes (de Melo Pereira et al. 2019; Septiana et al. 2025; Salahuddin et al. 2025; Shah et al. 2025; Latief et al. 2025). For the wet process, the coffee cherries are peeled to remove the skin to obtain wet parchment beans (WPBs), and this is followed by dry (solid‐state) and/or wet (water‐submerged) fermentation to reduce the mucilage pulp. The fermentation can be done for 24–36 h (Rocha et al. 2024), and the WPBs are washed and dried to obtain dried parchment beans (HS, horned skin). For the semi‐wet process, fermentation and washing processes are skipped by directly drying the WPBs to obtain the HS; these HSs are dehulled to obtain dried GCBs (Bastian et al. 2021); the dry process, on the other hand, is done by directly drying the coffee cherries to obtain dried coffee cherries. The dried coffee cherries are dehulled to obtain GCBs (Elhalis, Cox, and Zhao 2022).

Cherries of Liberica and Excelsa have different characteristics from those of Arabica and Robusta. These differences should be taken into consideration in choosing the processing methods used in the area. Liberoid coffee beans generally have a lower yield (8%–10%) than Arabica (16%–17%) and Robusta (∼20%) (Randriani and Dani 2018); this represents a lower bean‐to‐pulp ratio. Liberica cherries are bigger than those of Arabica and Robusta due to their thicker pulp (Davis et al. 2022). Pulp and skin of the cherries are important determinants for the quality of the beans produced. In the dry processing of coffee beans, thick pulp prolongs the drying and often leads to the production of off flavors (Maman et al. 2021; Sulaiman et al. 2021). In the wet processing of Arabica, these parts are removed during depulping, fermentation, and washing; this results in clean HS coffee beans assisting the drying process (Bastian et al. 2021; Febrianto and Zhu 2023). On the other hand, Robusta coffee cherries have thinner pulp than Liberoid and Arabica; this makes Robusta beans suitable for efficient dry processing. These methods offer their own advantages and disadvantages in the processing of Liberoid coffee beans. Current processing is usually done based on local practice and often adjusted based on its suitability with the geographical condition on the area.

### 4.1 Geographical Conditions as Determinants of Coffee Bean Processing Methods

Coffee beans are typically cultivated and processed within the same region; consequently, geographical conditions strongly influence the choice of processing methods (Banti and Abraham 2021). This is related to the relative humidity and temperature of the environment, mainly affecting the drying duration (Phitakwinai et al. 2019). Detailed reports on Liberoid bean processing are mostly from Asia, particularly Indonesia and Malaysia (Table 1). Wet processing is uncommon for Liberoid coffee due to several factors. This method is common for Arabica coffee. Arabica plantations are often located in high‐altitude areas (>1000 m above sea level). The microclimate is often conditioned by low temperature and high humidity (Woldegebriel 2025). This prevents quick drying of the coffee beans. Thus, wet processing, which eliminates the skin and pulp of the cherries, is commonly done to assist quick drying of the beans. However, wet processing requires investment in machinery, mainly depulper and washer (Bizimungu et al. 2024; Djafar et al. 2025); furthermore, wet processing requires a significant amount of water (Irawan and Mclelan 2024). These requirements conflict with the major goal of Liberoid coffee development, which emphasizes adaptability to marginal lands where water availability is often limited. Consequently, more water‐efficient processing methods are preferable for sustainable production.

**TABLE 1:** Liberoid coffee beans processing and its effect on chemical composition and sensory properties.

Material	Origin/genetic/specific identity	Processing method	Methodology	Effects on chemical composition	Effects on sensory quality	References
Liberica	Kopendukuh, Kalipuro, Banyuwangi, Indonesia. No information on the identification of the variety/genotype was provided	Emerging method: wet process with anaerobic fermentation	Coffee cherries were processed by the full‐wash method (wet process). The fermentation was carried out anaerobically using carbonated water to submerge the wet parchment coffee beans (1:2 w/v). The fermentation was performed for 5 days. The drying was carried out for 14 days. The dried beans were then de‐hulled to obtain GCBs. The brewing of the RB was done by V60, Vietnam drip, and French press method. The roasting degrees were light and medium	The differences evaluated in the study were between light level RBs to the medium level RBs. Medium RBs had significantly higher concentrations of every volatile analyzed. Volatiles with high concentrations included pyridines, methyl‐pyrazine, acetic acid, 5‐methyl‐furfural, 2‐furansmethanol, and isovaleric acid	The brew of light level RBs was characterized by high caramel and dried fruit flavor. They had low smoky, roasted, and chocolate aroma/flavor and low bitter taste and aftertaste. The brew of medium level RBs had high smoky aroma, smoky flavor, and roasted and chocolate flavor. However, it had high bitter taste and aftertaste. These trends were similar in all brewing methods	Septiana et al. (2025)
Liberica	Tanjung Jabung Timur, Jambi, Indonesia. No information on the identification of the variety/genotype was provided	No information provided. The GCB was obtained from a local market	The GCB was roasted at 203°C for 12 min. The cooled RB was then ground to fine powder. Analyses of proximate, sensory, TPC, TFC, caffeine, CGAs, and antioxidant activity were carried out	The RB had TPC of 42.3 mg GAE/g, TFC of 8.43 mg QE/g, caffeine content of 0.9%, CGA content of 2.98 %, and IC_50_ of 72.12 ppm. It consisted of 17.61% protein, 10.17% fat, and 5.94% carbohydrate (DB)	Coffee brew of RBs had an SCA score of 77.7, considered as premium quality. The brew had excellent aroma, flavor, aftertaste, acidity, and mouthfeel. However, it suffered from low uniformity, clean cup, and sweetness (8 out of 10). This indicated that the beans were highly varied. Further, eight out of 10 in clean cup showed the possibility of defect/off flavors in the brew	Latief et al. (2025)
Liberica	Kluang, Johor, Malaysia. The clones were MKL 8, MKL 9, and MKL 10	Conventional: dry process	The coffee cherries were dried in a hot air oven 60°C until 8–10% MC. Roasting was done on a medium roast level. Extract for analysis was prepared by ultrasonic–assisted extraction (40–50°C, 15 min, 10,000 RPM) using 70% ethanol at 1:10 (w/v). Antioxidant activity (DPPH and FRAP), TPC, TFC, and chromatographic analyses were performed	The highest antioxidant activity was evaluated from the extract of MKL 9 (IC_50_ DPPH, 7.3 mg/mL) and MKL 10 (FRAP, 9.6 mM). The extract of MKL 8 showed the highest TPC (148.7 mg CGAE/g). No significant differences in TFC were observed between clones (29.8–32.8 mg RE/g). GCB extract of MKL 10 clone had the highest caffeine content (9.9%) than that of MKL 8 (4%) and MKL 9 (2.9%). The extract of RB from MKL 8 showed the highest TPC (113.5 mg CGAE/g) and TFC (25.2 mg RE/g)	Not evaluated	Salahuddin et al. (2025)
Liberica	Jasin, Melaka, Malaysia. No information on the identification of the variety/genotype was provided	Emerging method: wet process with modified fermentation method. The fermentation was done with the addition of pectinase	The wet processing method was used for the processing of coffee cherries. Depulped coffee cherries (wet parchment coffee) (20 g) were put in 0.035% (w/v) pectinase solution and incubated at room temperature. Fermentation was done for 4, 6, 8, and 10 h. After fermentation, the beans were washed and dried in an oven (60°C) until 10–12% MC. Analysis of pectin content, TPC, and antioxidant activity were performed	The use of pectinase on the fermentation produced GCBs with lower pectin content. The reductions varied from 4.6% to 38.11%. Fermentation for 8 h resulted in the highest pectin removal (38.11%). Fermented GCBs also showed an increase in TPC range from 12.86% to 16.03% compared to control. Fermentation for 4 and 10 h increased antioxidant activity of the GCBs (1.14%–2.14%, respectively). Fermentation for 6 and 8 h reduced the antioxidant activity of the GCB (0.48%–0.83%, respectively)	Not evaluated	Shah et al. (2025)
Liberica	Lipa Batangas, Philippines (300–500 m a.s.l.)	No information provided. The GCB was obtained from a local supplier	RB was prepared by roasting the beans at 207°C for 12 min. The beans were then ground and analyzed for their volatiles using GC‐MS	Furfuryl alcohol, pyridine, 2‐methylfuran, 2‐methylbutanal, and 2‐methylpyrazine were among the volatiles showing the greatest peak area (%). Monoterpenoid such as terpinene 4‐acetate and trans‐β‐ocimene were also observed	Not evaluated	Dimaano et al. (2024)
Liberica	Banyuwangi, East Java, Indonesia. No information on the identification of the variety/genotype was provided	Conventional: dry process	No information on beans preparation (processing method) was provided. Roasted beans were prepared at a medium level. The brewing was done using three different methods, namely V60, French press, and Vietnam drip. Chemical analysis included caffeine content and IC_50_. Sensory analysis was done utilizing QDA involving eight trained panelists. Arabica coffee with the same processing method was used as comparison	The RB had caffeine content of 0.95% (w/w). The IC_50_ of the RB was 28.4 ppm, lower than that of Arabica (33.8 ppm). IC_50_ of brewed coffee were in range of 13 – 27.14 ppm. The highest IC_50_ was observed in Vietnam drip method (27.14 ppm) followed by French press (22.7 ppm) and V60 (13.03 ppm)	Liberica coffee showed similarity on sensory profile despite different brewing methods. Smoky aroma, smoky flavor, roasty flavor, bitter taste, bitter aftertaste and jackfruit aroma were particularly high	Azizah et al. (2024)
Liberica	Poncokusumo, East Java, Indonesia. Three accessions of Liberica coffee in the experimental garden	Conventional: dry process. Emerging method: Wine process (anaerobic fermentation)	The coffee beans were prepared by sun drying the coffee cherries for 5 h after washing and sorting. Wine‐process was done by storing the cherries in impermeable plastic for 45 days. Periodical sun drying (3 h every 5 days) was done during the fermentation time until the moisture reached 12%. Sensory analysis based on SCA method was then done	Not evaluated	Dry‐processed beans generally scored higher than that of wine‐processed. Light‐ and medium‐roasted beans had better sensory characteristics than those of dark roasted. Dark‐roasted Liberica coffees were characterized by high intensity of bitterness, astringency, smokiness, and burnt and rubbery flavor. Light and medium roast of dry‐ and wine‐processed beans exhibited distinct fruity, sweet, and chocolate notes	Wafaretta et al. (2024)
Liberica,	Tanjung Jabung, Jambi, Indonesia. The coffee was obtained as a grade C coffee. The samples were identified as Libtukom (*Liberika Tungkal Komposit*) variety	Emerging method: digested method by fermentation using starter culture of *Alcaligenes* sp. and *Exiguobacterium indicum*	The beans fermentation was carried out in glass container using 1 kg of green beans. The starter culture was added at the start of fermentation. Fermentation was done for 24 and 48 h. The drying was done using a convection oven. The beans were then roasted at three different roasting degrees (light, medium, and dark). Analysis included proximate analysis, total phenolic content, antioxidant assay, and IR fingerprinting on bioactive compounds using HPLC. Sensory analysis was done using the SCA method. Untreated coffee was used for control	Treated RB (medium roast level) showed increased TPC compared to the untreated one (11.7 mg GAE/g to ∼13.5 mg GAE/g). However, the DPPH inhibition (IC_50_) was reduced from 34 ppm (untreated) to 27.3–33.4 ppm. There was a decrease in the caffeine content in fermented samples. For bioactive composition, fermented RB had higher concentrations of n‐*O*‐caffeoylquinic acid and n‐*O*‐feruloylquinic acid than control RB	Fermented coffee beans scored higher (>79) than the control (70–72.5) in terms of sensorial quality. Fermentation of the beans with *E. indicum* for 24 h (medium roasted) resulted in a significant improvement in fragrance, flavor, sweetness, and the mouthfeel	Tarigan, Aulia, et al. (2024)
Liberica,	Tanjung Jabung, Jambi, Indonesia. The samples were identified as Libtukom (*Liberika Tungkal Komposit*) variety	Emerging method: digested method by fermentation using starter culture of *Bacillus subtilis*	Beans fermentation was carried out in 1‐kg batch capacity. The starter culture was added in the start of fermentation. Fermentation was done for 24, 36, and 48 h. The beans were then washed after fermentation and dried using a convection oven. The beans were then roasted at three different roasting degree (light, medium, and dark). Analyses included proximate, TPC, TFC, caffeine content, CGAs, and volatiles. Sensory analysis was done using the SCA method. Untreated coffee was used for control	Fermented samples had higher TPC (46.2 GAE/g) and TFC (12.5 mg QE/g) to that of the control (42.21 mg GAE/g, 8.4 mg QE/g for TPC and TFC, respectively). Decreased level of caffeine was evaluated (0.82 and 0.86% for fermented and control, respectively). No significant changes in CGA were observed. Treated GCBs had lower protein, fat, and carbohydrate content than the control	Fermented coffee showed improved sweetness, aroma and overall quality. Fermented coffee scored 86 SCA score compared to control (<80)	Tarigan, Adriliana, et al. (2024)
Liberica	Tanjung Jabung, Jambi, Indonesia. The samples were identified as Libtukom (*Liberika Tungkal Komposit*) variety	Emerging method: digested method using novel stainless fermentation tank. The starter culture was *Bacillus subtilis*	GCB was first sterilized using gamma irradiation (2.5 kGy). Coffee beans were placed in bioreactor and mixed with inoculum solution (1:1 w/v) with the population of 10^7^ CFU/mL. Fermentation was done for 12 h at 37°C with homogenization at every 4 h. The fermented GCB was then sun‐dried until 11% MC. The RB was prepared by light, medium, and dark roast level. Analyses included proximate and sensory analysis. Fermentation without the use of bioreactors, and commercial Liberica civet coffee was used as comparison	The fermentation produced GCBs with lower fat (9.28%), protein (0.78%), and carbohydrate content (21.25%) compared to the control (10.17, 2.09, and 71.24 for fat, protein and carbohydrate content, respectively)	RB from bioreactor‐fermented GCBs produces coffee brew with lower sensory quality than that of fermented without bioreactor. The SCA score was 75 (light roast), 81.75 (medium roast), and 78.33 (dark roast) for bioreactor‐fermented RB compared to 86.3 (medium roast) for RB from non‐bioreactor‐fermented GCB	Sutrisno et al. (2025)
Liberica	Batu Pahat, Johor, Malaysia. No information on the identification of the variety/genotype was provided	No detailed information. Presumably similar to semi‐wet/honey processing	The coffee cherries were washed, and the beans and pulp were separated manually. The beans and pulp were then dried and ground. Proximate analysis was done on the samples. LC‐MS analysis was done to evaluate the non‐volatiles compounds	The wet beans of Liberica had contents of ash (3.42%, w/w WB), fat (4.28%, w/w WB), protein (11.96% w/w WB), fiber (11.83% w/w WB), and carbohydrate (8.12% w/w WB). The beans contained 2‐amino‐3‐methyl‐butanol, nigerose, scopolin, cis‐5‐CQA, 3‐O‐FQA, hydroxymobarbital, deoxymiroestrol, emmotin A, and complex of Trp‐Ile‐Lys peptide	Not evaluated	Ismail et al. (2024)
Liberica	East Tanjung Jabung Regency, Jambi, Indonesia	No detailed information. Green beans were fermented with *Lactobacillus plantarum* and *Leuconostoc mesenteroides*	Green coffee beans (1 kg) were inoculated with *L. plantarum* and *Lc. mesenteroides*. The fermentation was done for 48 and 72 h. Fermented beans were then rinsed and dried in the sun and oven	Fermented beans had lower caffeine content with the lowest (0.117%) for the beans fermented with *L. plantarum* and *Lc. mesenteroides*. The highest caffeine content was evaluated on unfermented beans (0.86%). No significant changes in CGAs were evaluated. Lower total flavonoid and antioxidant activity were evaluated, despite the increase in TPC	Fermented beans obtained from 3 days fermentation using combination of *L. plantarum* and *Lc. mesenteroides* resulted in the highest SCA score (87.6), significantly higher than that of unfermented (77.1). Significant improvements were evaluated on every sensory attribute	Tarigan et al. (2023)
Liberica	East Tanjung Jabung Regency, Jambi, Indonesia	No detailed information. Green beans were fermented with *Lactobacillus plantarum*	The coffee beans were mixed with 500 mL of inoculum solution of *L. plantarum*. Fermentation was done for 36 h. Fermented beans were then washed and dried in the sun for 6 h followed by oven‐drying 50–60°C for 3 days	There were increases in TPC (from 42.3 to 46.4 GAE) and total flavonoids (from 8.4 to 12.9 QE). Lower caffeine and chlorogenic acid content were evaluated. For proximate composition, fermented beans had lower protein, lipid, and carbohydrate content	Fermented beans had higher overall score (86.3) than those of unfermented (77.7). Higher scores on aroma, flavor, aftertaste, body, and balance were evaluated. Fermented beans also had more uniformity, clean cups, and sweetness	Latief et al. (2023)
Excelsa	Wonosalam, East Java, Indonesia. No information on identification of the variety/genotype was provided	Conventional: (dry process, semi‐wet process and wet process) emerging method: (anaerobic fermentation of red cherry in combination with dry process)	Dry processed coffee was prepared by sun drying the coffee cherries until dried. Semi‐wet processing was done by drying the depulped coffee parchment until dried. Wet process was done by the method of “*giling basah*.” Anaerobic fermentation was done based on local method, carried out by repeating the anaerobic fermentation of the coffee cherry and drying. This was repeated until the desired moisture content was reached. No specific information on the moisture content and the duration (fermentation, drying, anaerobic fermentation) was provided. Quality analysis was done using the arabica SCA cupping method. Flavor analysis was done using HS‐SPME‐GC‐MS. Bioactive analysis was done using HPLC	The concentration of CQA and alkaloids varied among the GCBs and RBs from different processes. Dry‐ and semi‐wet processed beans showed higher CQA and alkaloids content that of wine‐ and wet processed. All RBs showed high concentration of aldehyde, alcohol, carboxylic acid, pyridine, furan, and pyrazine volatiles. Compared to other treatments, wine‐processed RBs had the lowest volatiles concentration	All the treatments resulted in specialty‐grade coffee (SCA score of >80) as analyzed based on the arabica SCA method. Dry processed coffee showed higher quality in aroma, acidity, balance, and overall attributes than that of other processes. Ethyl isovalerate was found to be the compound responsible for jackfruit flavor	Herawati et al. (2022)
Liberica	Pakuwon experimental garden, Sukabumi, West Java, Indonesia. The samples were identified as varieties of Liberoid Meranti	Emerging method: starter culture fermentation	Liberica coffee beans were first peeled to obtain wet parchment beans. The beans were then fermented in polypropylene bags with the addition of starter culture. The starter culture was a consortium of *Lysinibacillus fusiformis* strain Ma‐Su CECRI 2, *Bacillus cereus* strain L77, *Bacillus subtilis* strain GL2, and *B. cereus* strain F4a, previously isolated from civets’ saliva. The fermentation was done for 4, 8, and 12 h. The resulting beans were then dried until 12% moisture. Liberica coffee beans prepared with semi‐wet process were used as the control. Analyses included caffeine content, protein, fat content, and sensory analysis	Fermentation produced GCB with lower caffeine and higher fat content than untreated beans. Protein contents were varied affected by the fermentation duration. Short duration of fermentation increased the protein content (from 15.5 to 15.9 g/100 g). Longer duration of fermentation reduced the protein content (from 15.5 to 12.9 at 8 h of fermentation and 14.7 g/100 g at 12 h of fermentation)	Fermentation using starter culture for 8 h resulted in the best quality coffee. This received an SCA score of 82 compared to other treatments such as control (77.63), 4 h fermentation (78.13), and 12h fermentation (80.25). The higher score was due to improved fragrance, flavor, acidity, mouthfeel, sweetness and aftertaste	Wibowo, Mangunwardoyo, Yasman, et al. (2021)

Abbreviations: CGA, chlorogenic acid; CGAE, chlorogenic acid equivalent; CFU, colony‐forming unit; CQA, caffeoylquinic acid; DB, dry basis; DPPH, 2,2‐diphenyl‐1‐picrylhydrazyl; FQA, feruloylquinic acid; FRAP, ferric reducing antioxidant power; GAE, gallic acid equivalent; GC‐MS, gas chromatography‐mass spectrometry; GCB, green coffee beans; HPLC, high‐performance liquid chromatography; HS‐SPME‐GC‐MS, headspace‐solid phase microextraction–gas chromatography–mass spectrometry; IC_50_, half maximal inhibitory concentration; IR, infra‐red; kGy, kilograys; MC, moisture content; QE, quercetin equivalent; RB, roasted beans; RE, rutin equivalent; RPM, rotation per minute; SCA, specialty coffee association; TPC, total phenolic content; TFC, total flavonoid content.

**FIGURE 1:**
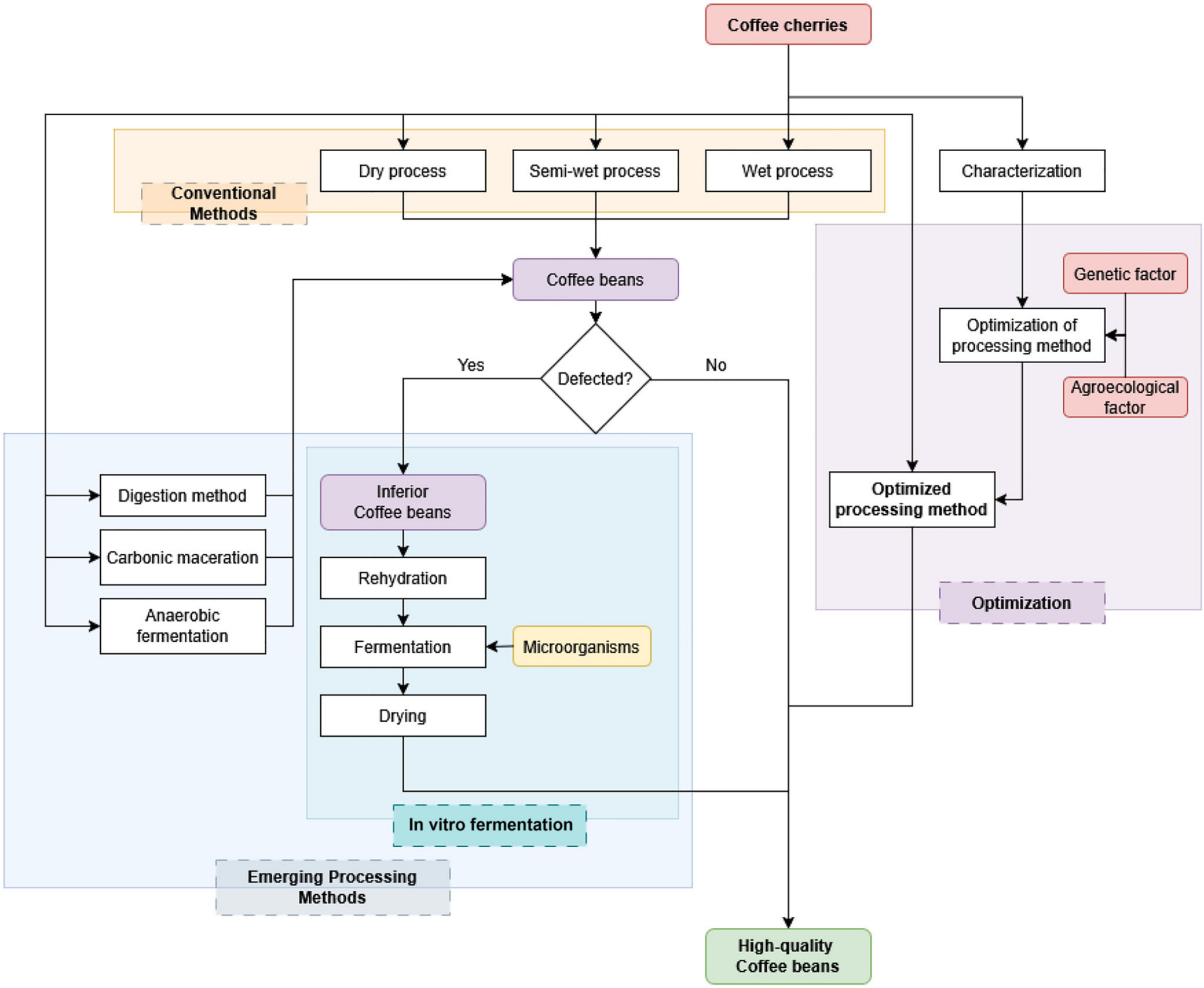
Schematic figure of coffee processing methods emphasizing conventional methods (yellow box), emerging processing method (EPM, blue box), and the use of in vitro fermentation (as a part of EPM) or the improvement of the quality of Liberoid coffee beans. Purple box showing the step of optimization of coffee processing method based on the genetic and geographical factors.

Dry processing methods are often used for the processing of Liberoid coffee beans, mainly due to economic and geographical reasons (Karim et al. 2019; Febrianto and Zhu 2023). Dry processing needs no additional treatment on the coffee cherries after harvesting. The cherries are directly dried under the sun, providing an efficient processing method for the farmers (Karim et al. 2019). Liberoid coffee plantations are often grown in geographical conditions similar to those of Robusta. Robusta is generally cultivated in a dry and low humid environment. This allows quick drying of coffee cherries due to high exposure to sunlight and high transfer of moisture between the beans and environment (Fauzi et al. 2024). While it works for Robusta coffee, dry processing of Liberoid coffee beans is less optimum. This is due to their thicker skin and pulp compared with Robusta coffee beans. In some areas, this dry processing is facilitated by cracking the cherries to expose the beans to allow quicker drying (Febrianto and Zhu 2023). However, this method is reported to have a high risk on sensory quality reduction due to fungi infestation from inefficient drying (Hagos et al. 2024). Considering these factors, wet processing could be the most suitable factor for Liberoid coffee processing; this is necessary to produce defect‐free and high‐quality coffee beans (Figure 1).

Marginal environments such as peatlands have been identified as target areas for Liberoid coffee cultivation (Waluyo and Nurlia 2017; Wibisono et al. 2024). Liberica coffee demonstrates greater environmental flexibility than Arabica or Robusta, as it can be cultivated in warmer, more humid, and less seasonal climates that fall outside the current ecological niche of the other two species (Wild et al. 2026). A comprehensive study on Liberica development in Rangsang Island (Indonesia) highlights the region's reliance on this commodity, which contributes to the sustainability of Liberoid coffee production (Wibisono et al. 2024). However, limited technical support in environmental conservation, infrastructure, technology adoption, and economic development has resulted in suboptimal processing conditions. To address these constraints, the deployment of grower‐friendly technologies tailored to local geographical conditions is urgently required. Specifically, water‐efficient wet processing machinery and assisted drying systems, such as solar and mechanical dryers, are essential to improve bean quality. Moreover, the optimization of processing methods should be guided by both genetic and environmental parameters (Figure 1). The effective implementation of such strategies will enhance product quality, strengthen market competitiveness, and support the long‐term sustainability of Liberoid coffee production.

### 4.2 Emerging Processing Methods on Liberoid Green Coffee Beans

Emerging processing methods (EPMs) of coffee beans are related to cherry preconditioning (Brioschi Junior et al. 2021; Pereira et al. 2022), modifying the fermentation method (Mulyara and Rahmadian 2021) with the use of specific microorganisms as a starter culture (da Mota et al. 2020; Martinez et al. 2021) and in vitro fermentation done on GCBs (C. Wang et al. 2020a, 2020b). EPMs are used either in combination with conventional methods (Martinez et al. 2021) or applied after the GCB is obtained (C. Wang et al. 2020a, 2020b). Previous studies have shown the application of EPM in Liberoid coffee beans, including wet processing combined with anaerobic fermentation (Septiana et al. 2025), wet processing with enzymatic fermentation (Shah et al. 2025), wine process (Wafaretta et al. 2024; Herawati et al. 2022), and wet processing combined with starter culture‐added fermentation (Wibowo, Mangunwardoyo, Yasman, et al. 2021).

In vitro fermentation of Liberoid GCBs has garnered increasing attention among producer‐level stakeholders, owing to its flexibility in raw material utilization, specifically the use of dried GCBs instead of high‐moisture WPBs, and the broad selection of microorganisms available for starter cultures (Tarigan, Adriliana, et al. 2024; Tarigan, Aulia, et al. 2024; Wibowo, Mangunwardoyo, Yasman, et al. 2021). The process typically begins with rehydration of the dried GCBs to restore moisture content, thereby creating optimal conditions for microbial growth. Selected microorganisms are then introduced, and fermentation is conducted over variable durations depending on the desired quality attributes. Following fermentation, the beans are dried to yield fermented GCB (Figure 1). Previous studies have demonstrated the potential of various microbial strains, which influenced the chemical composition (further discussed in Section 5.5.2.2) (Wibowo, Mangunwardoyo, Yasman, et al. 2021; Tarigan, Adriliana, et al. 2024; Tarigan, Aulia, et al. 2024). Notably, the application of in vitro fermentation to Arabica and Robusta beans has yielded promising improvements in sensory quality for mitigating defects in inferior or geographically constrained coffee beans (as discussed in Section 6.1.2.2) (C. Wang et al. 2020a, 2020b; Tang et al. 2021; N. Zhao et al. 2024). Accordingly, in vitro fermentation offers a promising approach for enhancing the quality of Liberoid coffee beans currently in production.

## 5 Chemical Composition of Liberoid Coffee Beans

### 5.1 Proximate Composition

The proximate composition of Liberoid, Arabica and Robusta coffee beans exhibits notable differences (Table 2). The protein content of Liberoid beans is comparable to that of Arabica and Robusta (14.1–14.7 g/100 g DB, dry basis). However, Liberoid beans contain a higher carbohydrate content (72.3 g/100 g DB) than Arabica (72.2 g/100 g DB) and Robusta (68.0 g/100 g DB). Lipid content in Liberoid beans (9.55 g/100 g DB) falls between that of Arabica (10.1 g/100 g DB) and Robusta (8.74 g/100 g DB). Among the three, Liberoid beans possess the lowest ash content (3.71 g/100 g DB) (Vasconcelos et al. 2007; Ismail et al. 2013; Dong et al. 2017; Gómez‐Merino et al. 2018; Saputri et al. 2020; Prakash et al. 2022).

**TABLE 2:** Chemical composition of Liberoid, Arabica, and Robusta coffee beans.

Chemical composition	Liberoid	Arabica	Robusta
Proximate composition (g/100 g DB)			
Moisture	12.54^a^	11.5^b^	10.4^c^
Protein	14.42^a^	14.1^b^	14.72^d^
Lipid	9.55^a^	10.1^b^	8.74^e^
Carbohydrate	72.32^a^	72.2^b^	68^d^
Ash	3.71^a^	4.7^b^	4.0^f^
Alkaloids (g/100 g DB)			
Caffeine	0.94^g^–1.24^g^	1.19^i^	2.14^d^
Trigonelline	0.56^h^–0.67^h^	0.92^i^	0.63^d^
Phenolics (mg/g)			
3‐*O*‐Caffeoylquinic acid (mg/g)	7.5^j^	3.64^k^	5.07^k^
4‐*O*‐Caffeoylquinic acid (mg/g)	10.7^j^	5.28^k^	6.78^k^
5‐*O*‐Caffeoylquinic acid (mg/g)	47.3^j^	37.9^k^	42.3^k^
3‐*O*‐*p*‐Coumaroylquinic acid (mg/100 g DB)	37.6^l^	6.53^l^	11.8^l^
4‐*O*‐*p*‐Coumaroylquinic acid (mg/100 g DB)	2.93^l^	7.65^l^	6.2^l^
5‐*O*‐*p*‐Coumaroylquinic acid (mg/100 g DB)	21.9^l^	52.3^l^	21.9^l^
*p*‐Coumaroylquinic acid (g/kg DB)	0.58^l^	0.67^l^	0.40^l^
As total CGA (g/100 g DB)	7.6–9.8^l^	5.20–6.70^l^	5.80–8.40^l^
Organic acids (mg/g)			
Acetic acid		3.20^j^	
Lactic acid		0.37^j^	
Citric acid	DNQ^m^	12.1^j^	3.91^d^
Quinic acid	DNQ^m^	6.0^j^	1.41^d^
Malic acid	DNQ^m^	4.4^j^	3.91^d^
Oxalic acid			0.03^d^
Succinic acid	DNQ^m^	1.61^j^	0.73^e^
Tartaric acid			1.36^e^
Formic acid			1.18^e^

Abbreviations: CGA, chlorogenic acid; DB, dry basis; DNQ, detected but not quantified.

^a^ Liberica coffee beans sold in Malaysia (Ismail et al. 2013).

^b^ Brazilian Arabica bean (Vasconcelos et al. 2007).

^c^ Java Robusta bean (Saputri et al. 2020).

^d^ Dry‐processed Robusta coffee bean from India (Prakash et al. 2022).

^e^ Robusta coffee bean from China (Dong et al. 2017).

^f^ Coffee bean samples from Mexico, Brazil and Vietnam (Gómez‐Merino et al. 2018).

^g^ Wild Liberica bean (Campa et al. 2005).

^h^ Wild Liberica bean (Campa et al. 2004).

^i^ Average of 54 coffee bean samples from nine different regions in Ethiopia (Sualeh et al. 2020).

^j^ Wet‐processed Arabica coffee bean, Castillo variety, average of two harvest sessions (Echeverri‐Giraldo et al. 2024).

^k^ Arabica and Robusta beans from various sources (Badmos et al. 2019).

^l^ Commercial bean samples from various origins (Gutiérrez Ortiz et al. 2019).

^m^ Liberica green beans (Hanifah et al. 2025).

Comprehensive characterization and quantification of macronutrients, particularly carbohydrates, proteins, and lipids, have been extensively reported for Arabica and Robusta beans (Fischer et al. 2001; Dong et al. 2015; Kulapichitr et al. 2019; Mehari et al. 2019). Detailed analysis on polysaccharides profile of Arabica coffee beans (variety of Bourbon, Catimor, Catuai, Caturra, Geisha, Java, KP423, Pacamara, and Typica) has been done by Lin et al. (2025). Lipid composition has been reported by Várady et al. (2024). However, no detailed study on these components of Liberoid coffee has been reported. For Liberoid beans, lipid profiling has identified the presence of palmitic, oleic, linoleic, α‐linolenic, and arachidic acids, although quantitative data remain unavailable (Junio et al. 2025). The relatively high carbohydrate and protein content in Liberoid beans may promote the enhanced formation of Maillard reaction products during roasting, contributing positively to flavor development (Murthy et al. 2019). The structural composition of polysaccharides, proteins, and lipids is important in determining coffee sensory quality. For example, arabinogalactans, galactomannans, and linoleic acid are important components determining the mouthfeel (later discussed in Section 6.2.3). Nonetheless, detailed compositional analysis of carbohydrates, proteins, and lipids in Liberoid coffee beans is still lacking and warrants further investigation.

### 5.2 Bioactive Compounds

Methylxanthines and phenolic acids are the major bioactive compounds in Arabica and Robusta coffee beans (Badmos et al. 2019; Sualeh et al. 2020; Prakash et al. 2022). Similar occurrences of these compounds have been reported in Liberoid coffee beans including Liberica and Excelsa varieties (Campa et al. 2004, 2005; Gutiérrez Ortiz et al. 2019; Hanifah et al. 2025). The caffeine content of Liberoid beans ranged from 0.94 to 1.24 g/100 g DB, which was either lower than or comparable to Arabica (∼1.19 g/100 g DB), but consistently lower than Robusta (2.14 g/100 g DB). However, its concentration can be as high as 1.69% in roasted Liberoid coffee beans (Wibowo, Mangunwardoyo, Santoso, et al. 2021). Caffeine functions as a potent psychostimulant through antagonism of adenosine receptors, thereby modulating sleep‐inducing neurochemical pathways (Reddy et al. 2024; Chang et al. 2025). Its concentration is influenced by roasting intensity (Samsidar et al. 2025). Based on EFSA NDA Panel (2015), caffeine is generally regarded as safe when consumed in moderate amounts, typically below 400 mg per day for adults. Caffeine enhances alertness and cognitive performance; however, excessive consumption may induce anxiety and limit consumer preference, particularly for high‐caffeine Robusta brews (Liu et al. 2024). Given their lower caffeine content, Liberoid beans present a promising alternative for low‐caffeine coffee formulations.

Trigonelline levels in Liberoid beans (0.56–0.67 g/100 g DB) are lower than those in Arabica (0.92 g/100 g DB) but comparable to Robusta (0.63 g/100 g DB). Trigonelline has been associated with antioxidant and anticancer properties, contributing to the health‐promoting potential of coffee (Nguyen et al. 2024). Liberoid coffee beans had more chlorogenic acids (CGAs) compared to that of Arabica and Robusta (Table 2). Among seven CGAs evaluated, 5‐*O*‐caffeoylquinic acid and 4‐*O*‐caffeoylquinic acid were the most abundant in Liberoid beans, with concentrations of 47.3 and 10.7 mg/g DB, respectively, exceeding those reported for Arabica and Robusta. CGAs, particularly caffeoylquinic acids, are associated with various health‐promoting effects, including antioxidant and anti‐inflammatory properties (Chandimala and Atjoni 2025). However, these compounds also contribute to the bitter and astringent sensory profile of coffee (Hu et al. 2025).

Notably, Junio et al. (2025) identified methylliberine as a species‐specific alkaloid in Liberica beans cultivated in the Philippines. Methylliberine is a nootropic compound with reported vasodilatory effects and neuroprotective properties, including mitigation of oxidative stress in the brain. Acute intake has been linked to enhanced concentration and mood regulation, with minimal impact on cardiovascular parameters (La Monica et al. 2023). The co‐occurrence of methylliberine and low caffeine content in Liberoid beans may represent a unique compositional advantage, offering the characteristic “awake” effect of coffee with reduced health risks associated with excessive caffeine consumption.

### 5.3 Organic Acids

Organic acids are key quality‐determining compounds in coffee beans, contributing significantly to the sensory profile, particularly acidity, across different *Coffea* species. Arabica beans typically exhibit higher concentrations of organic acids than Robusta, resulting in a more pronounced acidic taste. The predominant acids in Arabica include citric (12.1 mg/g), quinic (6 mg/g), malic (4.4 mg/g), acetic (3.2 mg/g), succinic (1.6 mg/g), and lactic (0.37 mg/g) acids (Echeverri‐Giraldo et al. 2024). In contrast, Robusta beans are characterized by lower overall acid content, with malic and citric acids being the major contributors (∼3.91 mg/g) (Prakash et al. 2022). Although quantitative data are currently unavailable for Liberoid coffee beans, qualitative analyses have identified the presence of malic, citric, succinic, and quinic acids (Hanifah et al. 2025). Further quantification and profiling of organic acids in Liberoid beans are warranted to elucidate their contribution to sensory attributes and potential health benefits.

### 5.4 Volatiles

**FIGURE 2:**
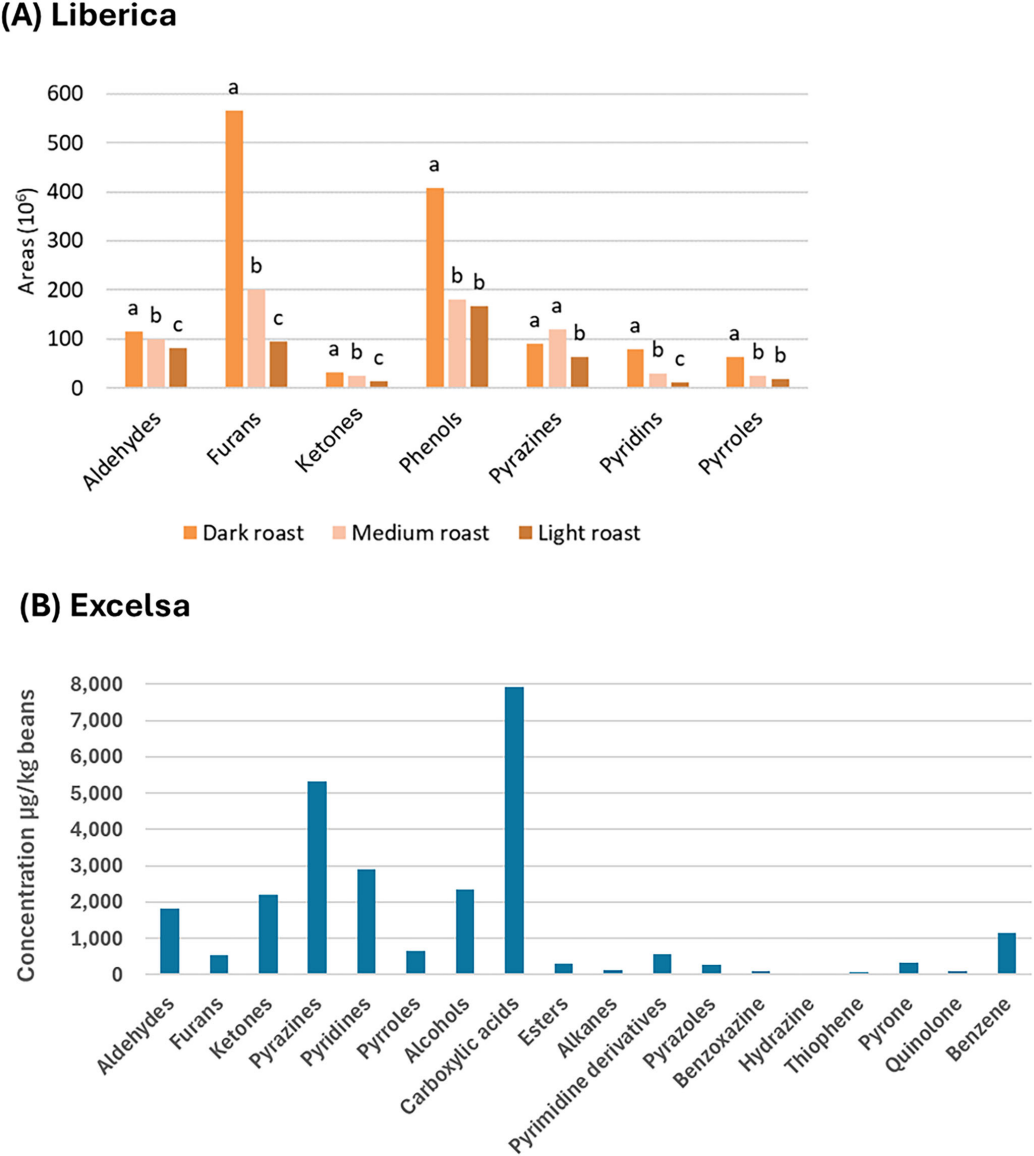
(A) Bar graph of the volatiles of the Liberica (variety LiM/Meranti) roasted coffee beans obtained from different roasting treatments. (B) Bar graph of the volatiles of the Excelsa roasted coffee beans obtained from wet process wet hulling methods. Data (A) were obtained from Wibowo et al. (2024). The publication was distributed under the Creative Commons Attribution 4.0 International License. Data (B) was derived from the published paper of Herawati et al. (2022). The published version was distributed under the Creative Commons Attribution License (unspecified version). Original data were in tabular form. Derivatization to the bar graph was carried out using Microsoft Office 365. Different letters above the graph in (A) show a significant difference (*p* < 0.05).

Volatile profile of Liberoid coffee beans showed a complexity similar to that of Robusta and Arabica. In roasted beans (RBs) of Liberica, volatiles spanning several chemical classes, including aldehydes, ketones, furans, phenols, pyrazines, pyridines, and pyrroles, were identified (Figure 2A) (Wibowo et al. 2024); dominant compounds included acetaldehyde, 2‐methylbutanal, 2‐methylfuran, 2‐furanmethanol, 1‐acetoxy‐2‐butanone, 4‐vinylguaiacol, methylpyrazine, 3‐pyridinemethanol, 3‐ethenylpyridine, and 1‐ethyl‐2‐formylpyrrole. For Excelsa RBs, Herawati et al. (2022) reported a broader spectrum of volatiles, encompassing aldehydes, alcohols, carboxylic acids, esters, ketones, pyridines, pyrimidine derivatives, furans, pyrazines, pyrazoles, pyrroles, benzoxazines, hydrazines, thiophenes, pyrones, quinolones, and benzenes (Figure 2B); among these, 17 compounds were identified as key aroma contributors, including five pyrazines associated with hazelnut, roasted, chocolate, earthy, and smoky notes. Notably, Excelsa coffee powder also contained volatiles typically associated with Arabica beans, such as acetic acid, ethyl isovalerate, furfural, and 5‐methylfurfural, which impart fruity, sweet, and vinegar‐like aromas (Herawati et al. 2022; Zhai et al. 2024; Taiti et al. 2025). Elevated concentrations of carboxylic acids in Excelsa further underscore its sensory resemblance to Arabica. Pyrazines, pyrroles, pyridines, and carboxylic acids are among the most influential volatiles differentiating Robusta and Arabica profiles: high pyrazine levels are characteristic of Robusta, whereas Arabica beans typically exhibit greater abundance of pyrroles, pyridines, and carboxylic acids (Zhai et al. 2024; Taiti et al. 2025).

Recent research has sought to isolate specific volatile markers that differentiate these varieties. In a study of 60 samples of coffee beans, Kiefer et al. (2026) identified variety‐specific markers: roasted Arabica beans were associated with furfural and 1‐hydroxy‐2‐propanone, while Robusta was linked to methylpyrazine and trimethylpyrazine. Liberica, by contrast, showed a high association with isovaleric acid, pyridine, and 1‐pentyl‐1H‐pyrrole. However, the authors highlighted that terroir factors, such as geographical conditions and processing methods, significantly influence the volatile composition of RB. This signifies the importance of environmental and post‐harvest variables in shaping the quality of Liberoid coffee beans (as discussed in Section 4.2). Consequently, further studies employing a more diverse range of samples obtained from diverse origins and processing conditions (both post‐harvest and roasting) are needed to better understand the distribution of volatile markers relative to these factors.

### 5.5 Effects of Coffee Genetics and Processing Methods

#### 5.5.1 Effect of Genetics on Chemical Composition of Liberoid Coffee Beans

Genetic factors play a pivotal role in shaping the chemical and biological properties of coffee beans, thereby influencing overall quality attributes (de Melo Pereira et al. 2019). The impact of genotypes on the chemical composition of Arabica and Robusta beans has been extensively investigated. In contrast, studies on the chemical composition of Liberoid coffee beans remain limited. As a result, establishing direct correlations between genetic information and chemical traits in Liberoid beans is currently constrained by insufficient data.

Genetic architecture in coffee plants is responsible for the formation of chemical compounds in the beans. Genome‐wide association studies on Arabica coffee showed that the production of lipids and diterpenes, a significant sensory quality contributor in Arabica beans, was influenced by the occurrence of specific genes. For example, kahweol production is dependent on the occurrence of Cc02_g33380 gene in S2_45775221 SNP vicinity (Sant'Ana et al. 2018). However, this study also revealed a complex relationship between genes to regulate the formation of specific compounds. Cafestol and kahweol are regulated by genes responsible for various enzymes, such as flavin‐containing monooxygenase, cytochrome P450 704, LCAS, triosephosphate isomerase, dihydrolipoyl dehydrogenase, momilactone A synthase, acyl‐CoA N‐acetyltransferases, and TATA‐binding protein‐associated factor 172 (Sant'Ana et al. 2018). Genome‐wide analysis of Liberica coffee has recently been reported (Melia et al. 2025); however, no direct correlations were established between genetic data and bean quality attributes. Given the compositional complexity of Liberoid coffee, which poses challenges for chemical characterization, comprehensive studies on the chemical profiles of beans derived from distinct Liberoid genotypes remain urgently needed. Such research is essential to elucidate genotype–chemotype relationships and support quality‐driven breeding and processing strategies.

A recent available report on Malaysian Liberoid coffee showed that variation under the same species resulted in slight variations of bioactive contents. Evaluation on three Liberica coffee clones (MKL 8, MKL 9, and MKL 10) showed that the flavonoid content of the GCB was similar (32.8, 29.8, and 32.8 mg RE/g for MKL 8, MKL 9, and MKL 10, respectively), and on the other hand, their phenolic content was slightly different (148.7, 138.1, and 121.7 mg CGAE/g for MKL 8, MKL 9, and MKL 10, respectively) (Salahuddin et al. 2025); these results agree with previous reports on other tropical crops of economic importance such as cocoa. Febrianto and Zhu (2023) reported that in cocoa, the differences in the species level (*Theobroma cacao*, *Theobroma grandiflorum*, *Theobroma bicolor*, and *Theobroma subincanum*) led to the differences in the profile of bioactive compounds (e.g., theacrine was found in *T. grandiflorum* and *T. subincanum* beans, but not in that of *T. bicolor* and *T. cacao*). This species‐specific metabolic difference is consistent with recent findings; Dai et al. (2026) found that each genetic of coffee (Arabica, Robusta, and Liberica) cultivate unique rhizosphere microbial communities and soil metabolite profiles, even under identical management conditions. Such host‐driven specialization at the root–soil interface likely provides the physiological foundation for the distinct bioactive profiles observed across different species. On the other hand, genotypes of the same species (e.g., different clones in the same *T. cacao* species) had similar bioactive composition, but in different concentrations (Febrianto and Zhu 2022).

Current cultivation of Liberoid coffee involves a limited number of clones/genotype/varieties; these include MKL 8, MKL 9, MKL 10, Meranti 1, Meranti 2, and Libtukom (*Liberika Tungkal Komposit*, composite of Liberica Tungkal) for the Liberica variety (Wibowo, Mangunwardoyo, Santoso, et al. 2021; Tarigan, Adriliana, et al. 2024; Tarigan, Aulia, et al. 2024; Salahuddin et al. 2025), but no report is yet available for the Excelsa variety. This is due to limited effort in plant breeding for the development of Liberoid coffee. An effort to classify the current cultivated Liberoid genotypes is urgently needed. This is essential to produce quality‐targeted planting material. However, it should be noted that the expression of genetics is significantly correlated to agronomical practices, environmental conditions, and stress factors (drought, pest and disease, pesticides, and fertilization). Hence, genetic and environmental variations in chemical composition on Liberoid coffee beans are expected.

#### 5.5.2 Effects of Postharvest Processing on Chemical Composition of Liberoid Coffee Beans

##### 5.5.2.1 Conventional Methods

Commercial Liberica coffee beans are predominantly dry‐processed. Among the evaluated samples (Table 1), RB exhibited modest variation in caffeine content, ranging from 0.86% to 0.95% (Azizah et al. 2024; Tarigan, Adriliana, et al. 2024; Latief et al. 2025). To date, no comprehensive comparative study on conventional processing methods has been reported for Liberica; however, such data are available for Excelsa coffee (Herawati et al. 2022). Processing methods that retained the skin and/or mucilage, namely dry and semi‐wet techniques, yielded GCBs with elevated levels of caffeoylquinic acid (CQA), trigonelline, and caffeine compared to wet processing. This enhancement is likely due to reduced leaching during minimal water contact with demucilaged parchment beans. Volatile profiling revealed that dry and semi‐wet processed GCBs contained a broader spectrum of compounds, including alcohols, carboxylic acids, esters, ketones, alkanes, pyridines, pyrazines, and benzenes, relative to wet‐processed counterparts (Herawati et al. 2022). While this diversity may contribute to a more complex flavor profile, certain volatiles were associated with off‐flavor notes, potentially compromising overall sensory quality.

**FIGURE 3:**
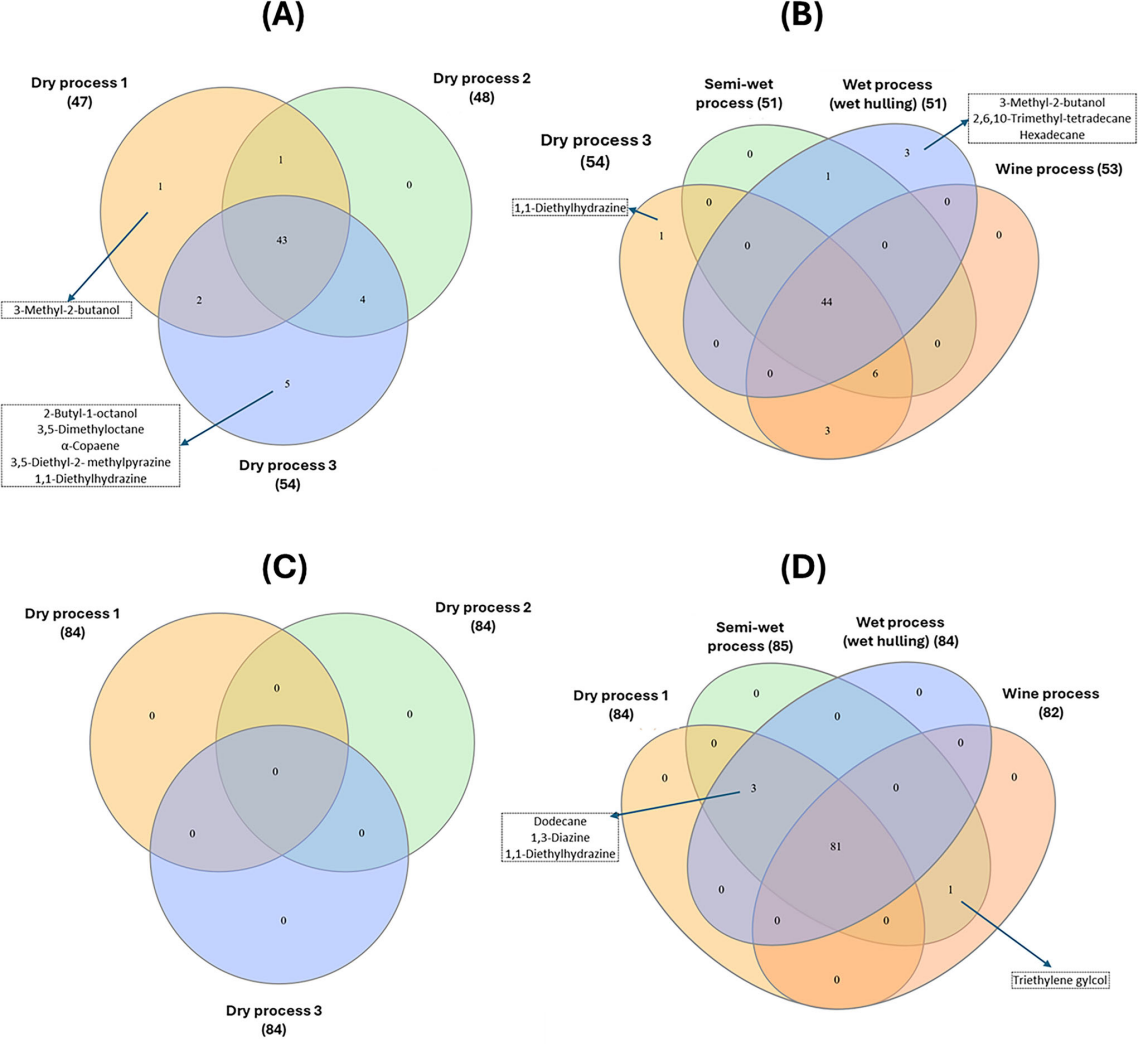
(A) Venn diagram of the volatiles of the Excelsa green coffee beans obtained from different dry‐process treatment. (B) Venn diagram of the volatiles of the green coffee beans obtained from dry, semi‐wet, wet and wine process treatment. (C) Venn diagram of the volatiles of the roasted coffee beans obtained from different dry‐process treatment. (D) Venn diagram of the volatiles of the roasted coffee beans obtained from dry, semi‐wet, wet and wine process treatment. Numbers in bracket following the type of processing used represent the number of volatiles identified on respective treatment. Volatile compounds inside the box were the unique volatile/s observed in the respective treatment. Data were derived from the published paper of Herawati et al. (2022). The published version was distributed under the Creative Commons Attribution License (unspecified version). Original data were in tabular form. Derivatization to Venn diagram was done using a platform of https://www.interactivenn.net/.

The impact of conventional processing methods on the volatile composition of Excelsa GCB and RB has been characterized by Herawati et al. (2022) (Figure 3). Volatile profiles of GCBs differed markedly depending on the processing technique employed (Figure 3A,B), as evidenced by the presence of unique compounds. For example, dry process 3 yielded volatiles such as 2‐butyl‐1‐octanol, 3,5‐dimethyloctane, α‐copaene, 3,5‐diethyl‐2‐methylpyrazine, and 1,1‐diethylhydrazine, whereas wet processing produced distinct compounds including 2,6,10‐trimethyl‐tetradecane and hexadecane, which were absent in beans subjected to other methods (Herawati et al. 2022). Although processing significantly influenced the volatile profile of GCBs, the type of volatiles detected in RBs remained relatively consistent across methods. Variations in RBs were primarily observed in the concentration of volatiles rather than their qualitative presence. This suggests that volatiles formed during pre‐roasting stages may be largely lost during roasting, likely due to their low initial concentrations.

The thin pulp layer characteristic of Excelsa cherries may facilitate efficient drying, thereby minimizing the accumulation of off‐flavor volatiles in GCBs; this anatomical feature could contribute to improved volatile retention and flavor quality during processing. The pulp of coffee beans affects the processing outcomes by several mechanisms. It is an important substrate for microorganisms during cherry and bean fermentation (Febrianto and Zhu 2023). Delayed depulping after harvesting allows the pulp to be a medium for the growth of yeast, mesophilic, and lactic acid bacteria, including *Saccharomyces cerevisiae*, *Hanseniaspora* sp., *Lactiplantibacillus plantarum*, *Staphylococcus* sp., *Weisella cibaria*, *Candida* sp., *Leuconostoc* sp., and *Bacillus subtilis* (Elhalis, Cox, and Zhao et al. 2022). The fermentation of the pulp by these bacteria produces various metabolites such as alcohols, organic acids, and volatiles (Elhalis, Cox, and Zhao 2022). However, sugar‐rich coffee pulp is prone to fungal contamination; this especially occurs in aerobic conditions such as during cherry storage (after harvesting) and drying (Maman et al. 2021). Fungal contamination (e.g., *Aspergillus* sp. and *Penicillium* sp.) produces off‐flavor volatiles and harmful substances (mycotoxins) (Maman et al. 2021; Sulaiman et al. 2021). This significantly reduces the quality of the coffee beans obtained. In terms of pulp thickness, Liberica coffee cherries has thicker skin and pulp (3–6 mm thick) than Excelsa (∼2 mm thick) (Davis et al. 2022). This allows Excelsa cherries to be dried quickly, eliminating the possibility of cherry over‐fermentation and high occurrence of undesirable compounds. In contrast, the thicker pulp and skin of Liberica cherries offer more disadvantages for conventional processing methods such as dry and semi‐wet processing. Also, the thick pulp may slow down the drying process, allowing more extensive fermentation and fungal infestation of the cherries.

The utilization of more controllable drying methods for Liberica coffee needs further investigation. Mechanical drying should be considered a more efficient approach compared to sun drying for limiting microbial‐induced degradation. A previous study by Firdaus et al. (2023) found that using a hybrid drying chamber for Liberoid coffee produced beans of quality comparable to that of sun drying, but with a significantly shorter drying time. However, its application on farmer's level should be adjustable into more practical solutions, considering the smallholder status and their economic factors. For example, small‐scale solar‐powered drying houses are a feasible alternative due to their low operational costs and more flexible construction.

##### 5.5.2.2 Emerging Processing Methods

The EPMs have markedly influenced the chemical composition of Liberica coffee beans. Notably, EPM‐treated beans exhibited reduced caffeine levels compared to those processed conventionally. For instance, Tarigan, Adriliana, et al. (2024) reported that fermented GCB of Liberica contained 0.82% caffeine, which was slightly lower than the 0.86% observed in dry‐processed counterparts. A similar trend was observed in Excelsa beans, where wine‐process treated samples demonstrated significantly lower caffeine content than those subjected to dry or semi‐wet processing (Herawati et al. 2022). The “wine process” specifically, where coffee cherries undergo anaerobic fermentation (often combined with partially aerobic conditions) for an extended duration (1–6 weeks), is often comparable to the state of “over‐fermented” coffee beans. To some extent, the intensive degradation of lipids, proteins, and carbohydrates leads to the production of undesirable volatiles that contribute to the development of off‐flavors (Febrianto and Zhu 2023). In vitro fermentation, a representative EPM, also led to reductions in lipid, protein, and carbohydrate concentrations in GCBs (Wibowo, Mangunwardoyo, Yasman, et al. 2021; Tarigan, Adriliana, et al. 2024). Similarly, Kartika et al. (2026) found a reduction in the intensity of aliphatic C‐H stretching bands of Fourier transform infrared spectroscopy spectra obtained from in vitro lactic acid fermented GCBs, indicating lipid degradation. This compositional shift parallelled that of civet coffee, where protein degradation occurred via leaching and proteolytic activity during gastrointestinal fermentation (Marcone and Alrifai 2019). EPMs further modulated phenolic content and antioxidant activity, though the extent of these effects varied. Shah et al. (2025) demonstrated that pectinase‐assisted fermentation increased total phenolic content (TPC) from 12.86% to 16.03%. A mechanistic understanding of enzyme‐ and microorganism‐mediated transformations is therefore critical for optimizing EPMs and enhancing the quality of coffee beans.

**FIGURE 4:**
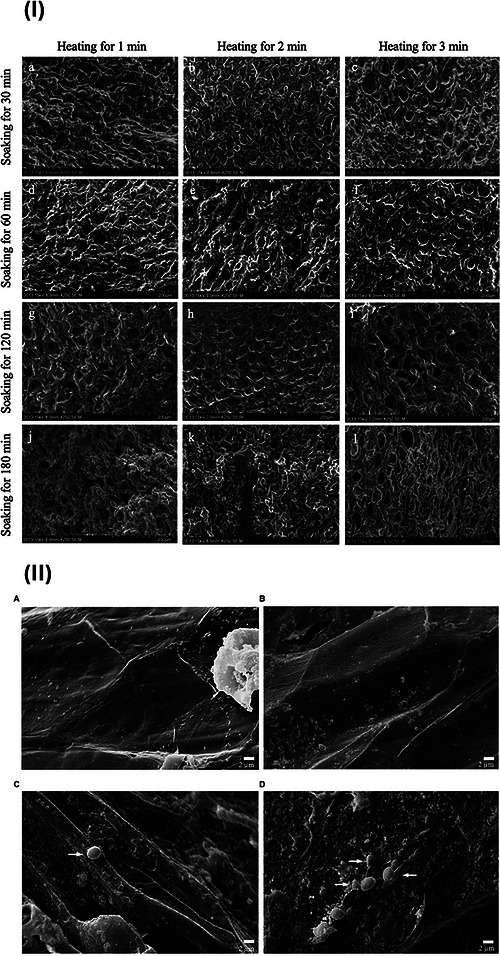
(I) Scanning electron microscope analysis of Kenyan Arabica green coffee beans obtained from different soaking and heating treatments. The figure depicted the changes in the microstructural conformation of coffee beans after prolonged soaking time, despite various heating treatment carried out (Dong et al., 2024). (II) A. Intact surface of control Arabica coffee bean at 0 h. B. Intact surface of control bean after 48 h of fermentation. C. Slightly degraded surface of *S. cerevisiae* CCMA 0543‐inoculated bean at 0 h (after inoculation). D. Heavily degraded surface of *S. cerevisiae* CCMA 0543‐inoculated bean after 48 h of fermentation. The figure was obtained from Dong et al. (2024) and Martinez et al. (2019). The published version was distributed under the Creative Commons Attribution License (unspecified version).

Most EPM methods focus on the optimization of fermentation process. This can be done by either prolonging the duration of fermentation or using external agents such as enzymes or microorganisms (Febrianto and Zhu 2023). Enzymatic and microbial fermentation in Arabica and Robusta coffee beans can decrease the concentrations of protein (as free amino acids), carbohydrates (as soluble sugars), and phenolics. Hence, a similar compositional trend is expected in Liberoid coffee beans. In vitro fermentation affects the chemical composition through multiple mechanisms. Soaking in incubation media triggers the microstructural changes in the coffee bean matrix (Figure 4) (Dong et al., 2024), as does the addition of specific microorganisms during fermentation (Martinez et al. 2019). These changes indicate the cellular degradation, particularly of carbohydrates and proteins as building block of cellular matrix. These structural changes then facilitate analyte diffusion between the incubation medium and the coffee beans. Furthermore, products of carbohydrate, protein, lipid, and phenolic degradation may react during roasting, contributing to the formation of flavor volatiles (Martinez et al. 2021).

EPMs increase the production of some important volatiles in Liberoid coffee beans. The study of Septiana et al. (2025) on Liberica coffee showed that the use of anaerobic fermentation method for 5 days on wet process produced RBs (medium level) with elevated concentrations of pyridines, methyl‐pyrazine, acetic acid, 5‐methyl‐furfural, 2‐furansmethanol and isovaleric acid, these compounds are typically associated with desirable aroma attributes in Arabica beans. In contrast, wine processing altered the domination of volatiles in roasted coffee beans from those of carboxylic acids, pyrazines, and pyridines (7913, 5315, and 2903 µg/kg beans as found in Liberoid wet‐processed coffee beans, respectively) to that of carboxylic acid, furans and pyrazines (3951, 2635, and 2352 µg/kg beans, respectively) (Herawati et al. 2022). Furans, particularly furfural and 2‐furanmethanol, are responsible for sweet, bready, almond‐like and nutty attributes; these attributes contribute more to the flavor profile of Robusta coffee. A significant reduction in carboxylic acids was also observed on wine‐process treated Excelsa coffee beans (Herawati et al. 2022). However, C. Wang et al. (2019) found that the use of lactic acid bacteria to ferment Arabica coffee beans increased the concentrations of organic acids, particularly lactic acid. Hence, the chemical changes induced by EPM are highly contingent upon the specific microbial or enzymatic agents employed, as well as the processing modality.

## 6 Sensory Properties of Liberoid Coffee

Sensorial properties are critical for coffee quality. Robusta and Arabica coffee have distinctly different sensorial profiles. Coffee brews of Robusta are characterized by their heavy‐body and bitter taste. On the other hand, Arabica coffee is characterized by their distinct acidity and aromatic notes (Sunarharum et al. 2014). Based on this classification, aroma/flavor, acidity, mouthfeel, and bitterness are four attributes separating the quality between Robusta and Arabica coffee. Sensorial quality of coffee is determined by the relationship of various quality aspects such as fragrance/aroma, flavor, mouthfeel, acidity, bitterness/sweetness, and aftertaste; optimal quality is associated with a balanced interplay among these parameters, and coffee beans exhibiting such equilibrium are highly valued by consumers for their flavor richness and complexity (Hall et al. 2022).

Liberica coffee, in its current dry‐processed form, is characterized by overtone jackfruit note, a notable fruity flavor. However, it suffers from high defect notes such as bitter, astringent, smoky, and burnt tastes. Azizah et al. (2024) reported that Liberica coffee beans from Banyuwangi of Indonesia were characterized by a high intensity of smoky, roasty, and bitter tastes. This agreed with Wafaretta et al. (2024) who found that dark roasted Liberica coffee beans had a high intensity of rubbery and other defect flavors. In contrast, Excelsa was reported to have favorable attributes including good aroma, balanced acidity, and overall flavor harmony (Herawati et al. 2022). Among the major types, Arabica is widely regarded as superior in quality, primarily due to its greater flavor complexity and aromatic richness (Hall et al. 2022).

Sensorial evaluation of coffee is usually done by standardized methods and certified/trained panelists. Sensory analysis based on Specialty Coffee Association (SCA) guidance is the most used method and commercially recognized (Baqueta et al. 2020); the protocol features the evaluation of fragrance, flavor, acidity (for Arabica), salt/acid (for Robusta), bitter/sweet (for Robusta), body/mouthfeel, aftertaste, sweetness, balance, clean cup, uniformity and overall, on the brew of medium‐roasted coffee beans (SCA 2022). However, there is still no SCA method specifically used for Liberoid. Liberoid is currently analyzed by using Robusta standard due to its more accommodating attributes (salt/acid and bitter/sweet). Sensory analysis methods suitable for Liberoid should be developed. Other methods such as quantitative descriptive analysis, CATA (check all that apply), hedonic test, and other customized methods are also used (Condelli et al. 2022; Na'im et al. 2024).

On the categorization of quality, the quality of coffee is divided into below specialty/fine (0–79.99) and specialty/fine (>80). Based on the occurrence of defects, below specialty coffee is divided into premium (70–79 points), exchange (60–69, with defects), below standard (50–59), less complexity with notable defects), and off‐grade (<50, significant defects) (SCA 2022). However, this commercial standard may not be used by all stakeholders of coffee trading. In the coffee value chain, growers and collectors predominantly rely on physical attributes, such as bean size, shape, and defect count, to determine market price. Exporters and importers typically incorporate both physical and sensory evaluations to assess quality and commercial value. However, quality criteria diverge further downstream. Industrial processors and end users, including cafés and individual consumers, place greater emphasis on sensorial attributes. Large‐scale industries often prioritize uniformity in flavor and appearance to ensure consistency across batches and product lines. In contrast, specialty cafés and artisanal markets tend to value uniqueness and customer experience, favoring beans with distinctive sensory profiles.

Robusta and Arabica coffee are distinctly different in various aspects. The former is characterized by a strong body, bitterness, and astringency, while the latter is acidic and flavorful. Robusta is preferred by users seeking instant, formulated, and caffeine‐oriented coffee products. On the other hand, Arabica is sought after by coffee enthusiasts, primarily for pleasure and its flavor profile. Currently, Liberica coffee is placed in between, possessing the characteristics of Robusta (high bitterness) and Arabica (fruity). In terms of distinctive characters, notable jackfruit flavor of Liberoid is its uniqueness. However, its potential is currently hindered by poor post‐harvest processing, leading to high non‐uniformity and off‐flavors. This divergence underscores the need for innovative processing technologies capable of tailoring coffee bean characteristics toward either uniformity or sensory differentiation. Such advancements are critical for meeting the evolving demands of diverse market segments.

### 6.1 Sensory Profiles of Liberoid Coffee Affected by Genetics and Processing

#### 6.1.1 Effects of Genetics

Information about the sensorial quality of Liberoid from different clones and genotypes is scarce. Previous reports on the differences between Liberica genotypes are available (as mentioned in Section 6.1.1). However, despite the available reports on different genotypes of Liberica such as MKL 8, MKL 9, MKL 10, Meranti 1, Meranti 2, and Libtukom (Salahuddin et al. 2025; Sutrisno et al. 2025; Wibowo, Mangunwardoyo, Santoso, et al. 2021), less is done on the comparative analysis of the sensorial properties. Libtukom variety, in the currently available form (dry‐processed), has an SCA score of 70–72.5 (grade 3 beans, medium roasted) with average score for each attribute of 7.25 (Tarigan, Aulia, et al. 2024). For Liberica Meranti variety, an SCA score of 77.6 was given, with the body/mouthfeel being the most notable attribute (Wibowo, Mangunwardoyo, Santoso, et al. 2021). These sensory outcomes are inherently linked to the physical transformation of the beans during processing; studies on Liberica beans from Nawangan, East Java, indicate that roasting profiles should consider beans' density, porosity, and dimensional expansion for its optimization (Amri et al. 2026). This suggests that the reported sensory scores for specific varieties like Libtukom or Meranti may be highly diverse due to less‐optimized roasting process. Standardized physicochemical characterization and development of optimum roasting profile are essential for meaningful comparison. However, there is no known Excelsa genotype that has been developed; as such, no comparison with Excelsa coffee beans from different genotypes, in terms of sensorial quality, can be made. Further study of comparative analysis should be done to evaluate the potential of available planting materials. This will inform the development of novel planting materials, especially for Excelsa, with superior sensory quality.

#### 6.1.2 Effects of Postharvest Processing on Sensorial Quality of Liberoid Coffee

##### 6.1.2.1 Conventional Methods

Conventional postharvest processing methods have shown limited effectiveness in optimizing the sensory potential of Liberoid coffee species, particularly Liberica. Despite its economic viability, the dry processing of Liberica beans has been widely criticized for yielding brews with low sensory quality, often dominated by smoky, bitter, and rubbery flavor notes (Azizah et al. 2024; Wafaretta et al. 2024). The highest reported sensory scores for dry‐processed Liberica beans were 77.6 (Wibowo, Mangunwardoyo, Yasman, et al. 2021) and 77.7 (Latief et al. 2025), placing them within the premium category. However, no documented evaluations have exceeded the SCA threshold of 80, which defines specialty‐grade coffee. In contrast, Excelsa beans have demonstrated compatibility with conventional processing methods, consistently producing brews with favorable sensory attributes, including pronounced aroma, balanced acidity, and overall flavor harmony; Excelsa samples achieved SCA scores above 80, qualifying them as specialty‐grade coffee (Herawati et al. 2022).

##### 6.1.2.2 Emerging Processing Methods

The use of EPM on coffee cherries or wet parchment beans affects the sensory quality of Liberoid beans in varied ways (Wibowo, Mangunwardoyo, Yasman, et al. 2021; Sutrisno et al. 2025; Tarigan, Adriliana, et al. 2024; Tarigan, Aulia, et al. 2024). Septiana et al. (2025) found that anaerobic fermentation for 5 days on WPB (after depulping) resulted in coffee with smoky aroma/flavor, chocolate note, and high bitterness (medium RB). However, light RB had more positive qualities such as high caramel and dried fruit flavor as well as low smoky flavor and bitterness. Wine process on Liberica coffee cherries (45 days of anaerobic fermentation combined with periodical sun drying) resulted in coffee with fruity, sweet, and chocolaty notes (light and medium roast). Dark RB had high bitterness and rubbery flavor. However, overall, the quality was inferior to that of dry‐processed ones. In contrast, wine process less affected the sensory quality of Excelsa coffee beans. The SCA score of wine‐process treated beans (82.33) was similar to that of dry‐, semi‐wet, and wet‐process treated beans (∼83).

The formation of new flavor compounds and elimination of off flavors/defects in Liberoid coffee beans are essential features for EPM. Wafaretta et al. (2024) demonstrated that wine‐process treated Liberica beans exhibited markedly different sensory profiles compared to their dry‐processed counterparts, despite both treatments being applied to depulped wet parchment beans from the same origin. For example, sensory profiles of dry‐processed beans (medium roasted) were characterized by caramel, nutty, dark, chocolate, mango, starfruit, grape, and orange peel; however, the flavor profile changed to jackfruit, passion fruit, spice, bitter, and astringent. On the other hand, in the same study, dark RBs (dry‐processed) were characterized by bitter, astringent, nutty, and earthy taste. Wine‐process treated beans on the other hand had chocolate, caramel, passion fruit, bitter, astringent, jack fruit, and nutty attribute, significantly different to its previous counterpart. This showed that some flavor/aroma might be newly formed or eliminated during the EPM processing. The outcome of such transformations is influenced by multiple factors, including the intrinsic properties of the raw material, the specific EPM protocol employed, and the selection of external agents such as microorganisms and enzymatic treatments.

EPMs by in vitro fermentation, so far, have provided positive results on the sensory quality improvement in Liberoid coffee beans. Tarigan, Aulia, et al. (2024), Tarigan, Adriliana, et al. (2024) showed that the fermentation of Liberica GCB using *Alcaligenes* sp. and *Exiguobacterium indicum* on Liberica Libtukom GCB for 24–48 h resulted in higher SCA score (>79) than control (70–72.5). *Alcaligenes* sp. and *E. indicum* are known to have a high proteolytic activity; in addition, *E. indicum* can produce broader carbohydrate‐active enzymes than *Acaligenes* sp. Beans fermented with *B. subtilis*, a species known for producing enzymes with strong carbohydrate‑active properties, used as a starter culture for Liberica Libtukom GCB for 24–48 h, achieved significantly higher sensory scores (86) compared with the control (<80). This fermentation improved the fragrance, flavor, sweetness, and mouthfeel of the brew (Tarigan, Adriliana, et al. 2024; Tarigan, Aulia, et al. 2024). Similarly, the fermentation of Liberica Meranti GCB with bacterial consortium (*Bacillus cereus* strain L77, *B. subtilis* strain GL2, *B. cereus* strain F4a, and *Lysinibacillus fusiformis* strain Ma‐Su CECRI) for 4–12 h, intended to mimic the production of civet coffee, increased the cupping SCA score from 77.6 to 82 (Wibowo, Mangunwardoyo, Yasman, et al. 2021). The studies also reported a reduction in sensory defects such as bitterness by the fermentation (Wibowo, Mangunwardoyo, Yasman, et al. 2021; Tarigan, Aulia, et al. 2024).

The improvement of the sensory quality of coffee beans as a result of microbial fermentation is contributed by several key mechanisms, including the production/elimination of directly related sensory compounds, the release of sensory compounds from the cellular matrix, the transfer of metabolites through diffusion in/out, and the production of compounds with the ability to release and mask flavor. The production/elimination of directly related sensory compounds includes the production of acids, alcohols, and esters from the degradation of mucilage as the result of yeast and lactic acid fermentation (Osorio et al. 2023) or the production of flavor precursors (Martinez et al. 2021). On the other hand, elimination mechanisms such as the degradation of methylxanthines (biodecaffeination and biotheobromination) and polyphenol oxidation may also contribute to the improvement of the sensory quality (e.g., decreasing the bitterness) of Liberica coffee. In line with the chemical changes (Section 5.5), various flavor precursors and volatiles are produced during fermentation. Amino acids and reducing sugars are produced through microbial enzyme degradation (Elhalis, Cox, and Zhao 2022; Lu et al. 2026). The occurrence of these metabolites in the beans significantly contributes to the formation of flavor volatiles during the roasting process through the Maillard reaction.

The internal migration of these volatile and non‐volatile metabolites into the beans has been reported. Osorio et al. (2023) found that the diffusion of metabolites such as acids, alcohols, and esters occurred during coffee bean fermentation, mainly from fermented mucilage into the endosperm of the beans, which significantly altered the sensory properties of the coffee. In the subsequent discussion, the authors underscored the critical role of fermentation duration, proposing that longer fermentation facilitates deeper metabolite penetration. This effect is likely attributable to increased structural breakdown of the beans, which lowers mass‑transfer resistance. Concurrently, structural degradation assisted the release of sensory‐related compounds from cellular matrices. These include phenolics (Shah et al. 2025), polysaccharides (Lin et al. 2025), and lipids (Fabella‐Garcia et al. 2025). These bioactives are sensory‐related, mainly providing tactile sensation of coffee (further discussed in Section 6.2.1).

Sensory‑related compounds can interact with one another. In coffee beans, strong volatiles such as pyrazines and aldehydes, which contribute to caramel and chocolate notes, can mask off‑flavors by dominating overall odor perception (El Hosry et al. 2025). Hence, additional flavor volatiles produced as metabolites may act in a similar manner. Astringency‑inducing compounds such as CQA negatively correlate with sweetness but enhance bitterness perception (Wei et al. 2025). When present at lower concentrations, their masking effects decrease, allowing acidity‑ and sweetness‑related notes to become more prominent and yielding a cleaner, brighter sensory profile (Echeverri‐Giraldo et al. 2024; Wei et al. 2025). The addition of water or another solvent during GCB incubation or fermentation can facilitate the transfer of analytes from the beans into the surrounding medium. The aqueous or incubation solution may extract various constituents from the beans, including flavor precursors, caffeine, volatiles, sugars, and organic acids (Cordoba et al. 2020; Bawornruttanaboonya et al. 2025). Structural degradation of the bean matrix, which facilitates the diffusion of metabolites into the beans (Osorio et al. 2023), may also operate in the opposite direction by enabling flavor compounds to diffuse from the beans into the fermentation solvent. This outward diffusion can help remove compounds associated with sensory defects. As the coffee industry increasingly seeks differentiated products, a comprehensive understanding of the mechanisms linking fermentation to sensory outcomes is essential. Such knowledge is critical for guiding fermentation processes toward coffee with targeted and desirable sensory profiles. Among the underlying mechanisms, the roles of flavor formation and defect removal during in vitro fermentation are essential for enhancing the quality of Liberoid beans, particularly when applied to lower grade coffee beans.

### 6.2 Improvement of Sensory Characteristics of Liberoid Coffee Using Targeted in Vitro Fermentation With Specific Microorganisms

The optimization of the sensory attributes of Liberoid coffee beans via in vitro fermentation has been previously documented (Section 6.1.2.2). This approach offers notable flexibility, particularly through the use of GCB as substrates in place of wet cherries or parchment coffee, and the incorporation of diverse microbial starter cultures; extensive applications of this technology to Arabica and Robusta varieties have been reported (Table 3). Overall, the careful selection of microbial strains and fermentation conditions is essential to achieve desirable sensory outcomes.

**TABLE 3:** Microorganisms and their roles in affecting the sensorial aspects of coffee beans and coffee products through in vitro fermentation.

Microorganisms	Sample	Methodology	Contribution				Reference	Applicability on Liberoid and possible outcomes
			Aroma/flavor	Acidity	Mouthfeel	Bitterness		
*Alcaligenes* sp. *Exiguobacterium indicum*	Liberica GCBs	Liberica GCB (1 kg) was added with 500 mL of inoculum solution. The fermentation was carried out for 48 h. The fermented GCB was then washed and dried in the oven until 12% MC. The roasting process was done in three different levels (light, medium, and dark). Proximate, HPLC, and sensory analysis was done in RB samples	Fermentation using starters increased the fragrance and flavor of the RB. Fragrance and flavor attributes were the highest on the samples treated with *E. indicum* for 24 h (medium roasted). The use of *Alcaligenes* sp. showed no significant changes compared to untreated samples	Fermentation reduced the acidity of the brew of the fermented RB	Fermentation increased the mouthfeel of the brew of fermented RB	A significant reduction in bitterness was observed on the brew of RB fermented with *Alcaligenes* sp. and *E. indicum*	Tarigan, Adriliana, et al. (2024)	Already done on Liberoid coffee
*Bacillus subtilis*	Liberica GCBs	Liberica GCB (1 kg) was inoculated with *Bacillus subtilis* and fermented for 24, 36, and 48 h. Fermented beans were washed and oven dried. The roasting was done in three levels (light, medium, and dark)	Fermented beans had slightly higher scores of aroma and flavor than unfermented ones	The acidity score of coffee from fermented beans was similar to that of unfermented	The body score of coffee from fermented beans was similar to that of unfermented	The sweetness score of coffee from fermented beans was higher (10) than that of unfermented (8)	Tarigan, Adriliana, et al. (2024)	Already done on Liberoid coffee
*Bacillus subtilis*	Liberica GCBs	Liberica GCB was sterilized with gamma radiation before placed in bioreactor and mixed with inoculum (10^7^ CFU/mL, 1:1 w/v). Fermentation was done for 12 h at 37°C. Fermented beans were then sun‐dried. Roasting process was done by light, medium, and dark roast level. Coffee beans fermented without bioreactors and commercial Liberica civet coffee were used for comparison	Fermentation without bioreactor resulted in higher aroma and flavor scores on medium roast (8.25 and 8.00, respectively) than that of bioreactor (7 and 7, respectively)	Fermentation without bioreactor had slightly lower acidity on medium roast (7.7) than that of bioreactor (7.75)	Coffee beans obtained from bioreactor fermentation had higher body score (7.90) than that of without bioreactor (7.25)	Coffee beans obtained from fermentation with and without bioreactor had similar sweetness score (10)	Sutrisno et al. (2025)	Already done on Liberoid coffee
*Lactobacillus plantarum*	Liberica GCBs	Liberica GCBs (1 kg) were mixed with 500 mL solution containing *L. plantarum*. The fermentation was done for 36 h. Fermented beans were then washed and dried. Roasting was done using cupping test standard (medium roast)	The score of aroma and flavor increased from 7.58 and 7.67 to 8.25 and 8.00, respectively	No significant changes in the acidity were observed. The score of acidity was similar for unfermented (7.77) and fermented beans (7.70)	The body of the coffee from fermented beans was slightly higher (7.90) than that of unfermented (7.67)	Bitterness attribute was not evaluated. Sweetness score increased from 8 (unfermented beans) to 10 (fermented beans)	Latief et al. (2023)	Already done on Liberoid coffee
*Lactobacillus plantarum* *Leuconostoc mesenteroides*	Liberica GCBs	Liberica GCBs (1 kg) were mixed with 500 mL solution containing *L. plantarum* and *Lc. mesenteroides*. The fermentation was done for 36 h. Fermented beans were then washed and dried. Roasting was done using cupping test standard (medium roast)	The use of *L. plantarum* resulted in coffee beans with higher aroma and flavor score. The use of *Lc. mesenteroides* lowered the aroma and flavor scores. The use of their combination resulted in higher scores of aroma and flavor	The use of single inoculum resulted in fermented beans with a lower acidity score (7.3–7.5) than that of the control (unfermented, 7.77). The use of combination of *L. plantarum* and *Lc. mesenteroides* resulted in a higher acidity score (8–8.08)	The use of single *L. plantarum* or *Lc. mesenteroides* resulted in a lower body score. The use of their combination resulted in higher score of body	The use of inoculum resulted in a higher score of sweetness (10) than that of unfermented (8)	Tarigan et al. (2023)	Already done on Liberoid coffee
*Saccharomyces cerevisiae*	Arabica GCBs	Arabica GCB was dehydrated in water (10 g/L) for 15 min at room temperature. The beans were then steamed for 10–30 min until 25% MC. The starter culture was then added (0.5–1.5 g/kg) and added with sucrose (0–50 g/kg). The fermentation was done in a sealed container for 18–70 h. The fermented GCB was then dried at 70°C until 12% MC. Untreated sample, commercial sample with low fruity attributes, and commercial sample with high fruity attributes were used as references	The treatment produced coffee brew with two distinct fruity characters such as low fruity and high fruity. The detailed character as follows: 1. Low fruity: complex spices, roasted, nutty and cocoa flavor 2. High fruity: Complex fruity, berry, vanilla, brown sugar, nutty, cocoa, sweet, and spices flavor Both low (3.17) and high (6.35) fruity samples had higher fruity flavor than that of control (1.85) and references (1.96)	Low fruity category produced coffee brew with lower acidity than control. High fruity samples had higher acidity than the control	Low and high fruity category produced coffee brew with lower mouthfeel than control	Low fruity category produced coffee brew with higher bitterness and lower sweetness than control. High fruity samples had higher sweetness and lower bitterness than control	Moreno et al. (2025)	The application of high fruity treatment would result in an increase of acidity, aroma and flavor but lower mouthfeel and bitterness
*Rhodotorula mucilaginosa* (treatment YR) *Wickerhamomyces anomalus* (treatment YW) *Enterococcus mundgtii* (treatment LAB)	Arabica GCB (wet‐processed) from Colombia and Kenya	Fermentation was carried out using 5 g of green beans added with 5 g of dry pulp and 240 mL of water in 500 mL flask. The fermentation was done for 72 h at 28°C. Analysis was done using HPLC, GC‐MS, and sensory analysis	Fermentation with starter culture for 24 h resulted in increased number and concentration of volatiles in Colombian GCBs, while Kenya GCBs on 72‐h fermentation. In Colombian RBs, fermentation using YW at 48 h resulted in the highest concentration of volatiles. The use of YR, YW, and YW+LAB for 24 and 72 h showed the highest concentration of volatiles in Kenyan RBs	Fermentation using starter culture increased the acidity on Colombian and Kenyan RBs. The use of YR and YW combined with LAB resulted in the highest acidity in Colombian and Kenyan RBs, respectively	The use of starter cultures (24 to 48 h fermentation) increased the mouthfeel of Colombian RBs. The use of them in Kenyan coffee resulted in reduced mouthfeel except for 72 h fermentation	The use of YW and YW + LAB increased the amount of CGAs in Colombian GCBs. Fermentation in Kenya GCBs for 24 h increased the CGA. Longer duration of fermentation reduced the amount of CGAs. No significant changes in caffeine content were evaluated between treatment and the control	N. Zhao et al. (2024)	The use of YW and YW + LAB as starter culture in medium duration of fermentation (48 h) would produce Liberoid coffee beans with increased aroma/flavor, acidity, mouthfeel, and reduced bitterness
*Saccharomyces cerevisiae* (Belgian ale) *Saccharomyces cerevisiae* (Tropical IPA) *Torulaspora delbrueckii* (Biodiva) *Saccharomyces cerevisiae* (71B) *Saccharomyces cerevisiae* (Sourvisiae)	Arabica GCB	The fermentation medium was water (70 g) for 71 g of GCBs. The initial starter culture population was 5.94 × 10^11^ CFU/mL. Fermentation was done for 72 h. The fermented GCB was then dried at 45°C for 4.5 h	All treatments showed a similar trend with increased sweet potato, floral, fruity, and nutty notes. The coffee‐like flavors were significantly lower than untreated sample	The treatment using Sourvisiae resulted in the brew of RB with higher level of acidity than the untreated RB. The use of Belgian ale, Tropical IPA, Biodiva and 71 B significantly reduced the acidity of the coffee brew	Not evaluated	All treatments other than Sourvisiae showed a reduced level of bitterness and an increased level of sweetness compared to that of untreated	Calderon et al. (2023)	The use of Sourvisiae would produce Arabica‐like Liberoid coffee. On the other hand, the use of Tropical IPA, Biodiva, and 71 B would be suitable to produce Robusta‐like Liberoid coffee
*Saccharomyces cerevisiae* *Candida parapsilosis* *Pichia guilliermondii* *Leuconostoc mesenteroides* *Lactobacillus plantarum* *Pediococcis pentosaceus*	Arabica GCBs (dry‐processed)	Arabica coffee beans (1300 g, 7.66% MC) were rehydrated by immersion in ultrapure water (4°C, 24 h). The hydrated GCB (54.69% MC) was sterilized at 100°C for 40 min). Fermentation was done by adding 5 mL starter culture to 30 g of GCB by spraying. The fermentation was done for 5 days at 30°C	More alcohol such as phenylethyl alcohol, glycerol and 2,3 butanediol were produced by *S. cerevisiae*. LAB produced more ethylene glycol, pinitol, arabitol, and 1‐monomyristin	The use of *S. cerevisiae* and LAB as starter culture increased acids concentration and types in the green beans	Not evaluated	The use of starter cultures reduced sugar content. The use of LAB and *S. cerevisiae* increased the concentration of phenolic acids	Kim et al. (2022)	The use of *S. cerevisiae* and LAB as starter culture would produce Liberoid coffee beans with increased aroma/flavor, acidity, and increased bitterness
*Aspergillus luchuensis* Inui (JCM 22239) *Aspergillus oryzae* (Ahlburg) Cohn var. *brunneus* Murakami (JCM 2059) *Mucor plumbeus* Bonorden (JCM 3900)	Robusta GCBs	Robusta GCB was soaked in water (1:2 ratio) for 24 h. The beans were then drained and autoclaved. After cooling, the GCB was then inoculated with 1 g of starter culture spores and mycelia. The incubation was done for 7 days at 30°C with daily agitation. Control was prepared in the same way but without inoculation	Fermented GCBs treated with JCM 2059 and JCM 22239 had an increased concentration of amino acids. This indicated improved Maillard reaction during roasting, resulting in increased amount of volatile reaction products. JCM 22239 especially had a high amount of reducing sugars, an important Maillard flavor precursor	Not evaluated	Not evaluated	Fermented GCB had lower concentration of phenolics and caffeine than that of control. This could be related to lower bitterness of the coffee brew	Tang et al. (2020)	The use of starter culture would produce Robusta‐like Liberoid coffee beans with strong Maillard‐related aroma/flavor and reduced bitterness
*Saccharomyces cerevisiae* MERIT *Pichia kluyveri* FROOTZEN	Arabica GCB (Semi‐wet processed)	Arabica GCB (1 kg) was immersed in 2.3 L deionized water and autoclaved for 30 min (100°C). Fermentation was done using starter as follows: 1. FSc, *S. cerevisiae* 2. FPk, *P. kluyveri*. 3. Control Fermented GCB was then dried in the oven until 10% MC	Treatment of FSc and FPk had an increased level of nutty, roasted and smoky aroma/flavor. The increase in pyrazines and pyridines concentration was observed from all treatments	All fermentation treatments produced brew of RB with decreased acidity level. Apparent reduction in organic acid level was observed from all fermentation treatments	Not evaluated	Not evaluated	C. Wang et al. (2020a)	The use of starter culture would produce Robusta‐like Liberoid coffee beans with strong Maillard‐related aroma/flavor and reduced acidity
*Saccharomyces cerevisiae* MERIT *Pichia kluyveri* FROOTZEN *Lactococcus lactis* subsp. *comprehensive cremoris* ATCC 19257	Arabica GCBs (semi‐wet processed)	Arabica GCB (1 kg) was immersed in 2.2 L deionized water and autoclaved for 30 min (100°C). Fermentation was done using starter as follows: *1*. FYCo, co‐culture *S. cerevisiae* + *P. kluyveri* 2. FLYCo, sequential inoculation of *Lc. Lactis* subs. *cremoris*, *S. cerevisiae* and *P. kluyveri*. 3. FL, *Lc. Lactis* Fermented GCB was then dried in the oven until 10% MC	FLYCo produced coffee brew with increased fruity, winey, and sweet aroma/flavor. Brew from FL and FYCo resulted in caramel, roasted, and nutty aroma. However, increased smoked flavor was also observed in the brew of FL and FYCo. The increase of furfural, ketones, aldehydes, and pyrazines were apparent in fermented samples	All fermentation treatments produced brew of RB with decreased acidity level	Not evaluated	Not evaluated	C. Wang et al. (2020b)	FLYCo would be suitable to produce Liberoid coffee with Arabica aroma/flavor. FYCo would be suitable to produce Liberoid coffee with Robusta characteristics
*Lactobacillus rhamnosus* HN001	Arabica GCB	The fermentation was done by submerged fermentation. One kg of coffee beans was boiled with 2.2 kg of water for 30 min. The starter culture was added with the concentration of 7.5 log CFU/mL. Sample FN was without sucrose addition. Sample of FS was added with sucrose (1% w/w). Incubation was done for 0‒72 h at 37°C. The fermented GCB was then dried in oven (45°C) until 7% MC	Fermented RB with treatment of FS and FN produced RB with improved volatiles concentration (based on total peak area percentage) compared to that of the control. Sensorially, FS treatment produced coffee brew with a higher intensity of roasted, caramel, burnt, and smoky aroma than that of control. FN treatment produced coffee brew with lower intensity of caramel, burnt and smoky aroma than control	Fermented RB with treatment of FS showed higher concentration of organic acids. This was in line with sensory analysis which showed elevated acidic intensity. Treatment FN had similar organic acids concentration to that of the control	Not evaluated	FN treatment produced coffee brew with higher concentration of sugar than that of the control. On the other hand, FS showed lower levels of sugar than the control. This indicated that FN had lower bitterness and FS had higher bitterness than control	C. Wang et al. (2019)	Treatment FN and FS would be suitable to produce Liberoid coffee with strong Robusta‐like aroma/flavor with increased acidity and sweetness.
*Yarrowia lipolytica*	Arabica GCBs	Arabica GCBs (800 g) were rehydrated with water at 4°C for 24 h before steamed at 80°C (40 min). The GCB was then inoculated with *Y. lipolytica* (10^5^ CFU/g bean). The fermentation was done at 30°C for 5 days. After fermentation, the beans were steamed at 100°C and then dried	The changes in flavors were indicated by the changes in the volatiles. There were increase in pyrazines, alcohols, terpenes, ketones, furanones, and phenols on the treated RB compared to that of untreated RB	Lower organic acid concentration was observed in fermented beans than that of untreated	Not evaluated	No changes in the phenolic acid concentration were observed between fermented and untreated samples	L. W. Lee et al. (2017)	The use of starter culture would produce Robusta‐like Liberoid coffee beans
*Rhizopus oligosporus*	Arabica GCB (semi‐wet processed)	The arabica GCB was prepared by soaking in water (20% w/v) at 4°C for 24 h. The rehydrated beans were then steamed at 80°C for 40 min. Solid state fermentation was done utilizing starter culture (10^4^ CFU/g) for 5 days. The resulting beans were steamed and oven‐dried at 70°C until 7% MC	The changes in flavors were as follows. 1. Light roast: increase in caramelly, buttery and smoky flavors. Reduction in spicy, sulfuryl, roast, and sweet flavor was observed 2. Medium roast: increase in roast, nutty, caramelly, and sweet flavors. Reduced levels of smoky flavors were observed 3. Dark roast: increase in caramel flavor. Reduced levels of nutty, roast, smoky, spicy, sulfuryl, and sweet flavors were observed	The fermentation reduced acidity. Organic acid concentration decreased from 53.59 (control) to 35.22 mg/g (samples)	Not evaluated	Not evaluated	L. W. Lee et al. (2016)	The use of starter culture would produce Robusta‐like Liberoid coffee beans

*Note*: The column of “Applicability on Liberoid and possible outcomes” was related to interpretative synthesis and suggestions by the authors of the currrent review based on the results of respective study.

Abbreviations: CGA, chlorogenic acid; CFU, colony‐forming unit; GC‐MS, gas chromatography–mass spectrometry; GCB, green coffee beans; HPLC, high‐performance liquid chromatography; LAB, lactic acid bacteria; MC, moisture content; RB, roasted beans.

#### 6.2.1 Microorganisms Targeting Aroma/Flavor

Fruity, floral flavor and acidic taste are considered desired in coffee brew (Moreno et al. 2025). Floral, flowery, and fruity aroma/flavor is often correlated with the occurrence of specific compounds including aldehydes, alcohols, esters, carboxylic acids, and terpenes (de Carvalho Neto et al. 2020). Some microorganisms such as *Pichia fermentans* and *S. cerevisiae*, produce these compounds, such as phenylethyl acetate (sweet, spicy, wintergreen aroma), methoxyisopropyl acetate (floral, honey, rosy, chocolate, and cocoa notes), D‐limonene (sweet, orange, citrus), phenethyl alcohol (floral, sweet, bready), and nonanal (green lemon peel, citrus melon rind), which contribute to the various desired flavors (de Carvalho Neto et al. 2020). The presence of these compounds in coffee is beneficial for producing Arabica‐like quality. Conversely, extended fermentation with *S. cerevisiae* enhanced the development of caramel‐like flavor notes, which are characteristics of and well‐suited to Robusta‐type coffee profiles. This was reported by C. Wang et al. (2020a, 2020b) who used *S*. *cerevisiae* MERIT and *Pichia kluyveri* FROOTZEN for in vitro fermentation of Arabica GCBs. The use of alcoholic beverages‐specific strain of *S*. *cerevisiae* (Belgian Ale, Biodica, Tropical IPA, 71B and Sourvisiae) produced coffee with sweet potato, floral, fruity and nutty notes (Calderon et al. 2023). Hence, choosing the appropriate strain and understanding the metabolic pathway of microorganisms are important for obtaining desired flavor formation in Liberoid bean fermentation.

Studies on microbial fermentation of Liberoid, Robusta, and Arabica coffee beans suggest at least two fundamental mechanisms underlying flavor development during fermentation. First, microbial activity promotes structural degradation of the beans, leading to compositional changes in key chemical constituents (see Section 5.5.2.2). Second, extensive hydrolysis of proteins and polysaccharides generates free amino acids and reducing sugars, which serve as precursors for Maillard reactions contributing to flavor formation. These compositional changes enhance the formation of Maillard‐derived flavor compounds during the subsequent roasting. These products, including pyrazines, pyrroles, alkylpyridines, acylpyridines, furanones, furans, oxazoles, and thiophenes, are predominantly associated with caramel, chocolate, roasted, nutty, fried, cocoa, earthy, and hazelnut notes (Cherniienko et al. 2022; Herawati et al. 2022; Zhai et al. 2024; Taiti et al. 2025). In parallel, microbial fermentation contributes to flavor development through the biosynthesis of volatile compounds, such as alcohols, esters, and aldehydes, via glycometabolic pathways and fatty acid catabolism (Zhang et al. 2023). For example, yeasts including *S. cerevisiae*, *Candida* spp., and baker's yeast can produce ethanol, hexanal, and phenylacetaldehyde (Guneser et al. 2022; J. Wang et al. 2024). Additionally, *Rhizopus* spp. are capable of synthesizing geranyl acetate, an ester terpenoid imparting rosy and fruity aroma notes (Londoño‐Hernández et al. 2017; Wu et al. 2018). Further exploration of microbial diversity is warranted to expand the fermentation toolkit, particularly for Liberoid coffee beans, which may benefit from tailored microbial interventions to achieve distinctive sensory profiles.

#### 6.2.2 Microorganisms Targeting Acidity

In vitro fermentation using lactic acid bacteria (LAB) has been shown to enhance the acidity profile of coffee, a sensory attribute particularly valued in Arabica varieties. LAB are widely employed in fermented dairy products such as yogurt and acidic beverages, where they contribute to sharp, tangy, and pleasant flavor notes (Ahansaz et al. 2023). This acidification effect is considered beneficial for improving coffee quality. The application of *L. plantarum* and *Leuconostoc mesenteroides* in Liberoid coffee has been reported (Latief et al. 2023; Tarigan et al. 2023); the use of their combination as a starter culture in Liberica GCB fermentation produced coffee brew with high acidity score (8–8.08) (Tarigan et al. 2023). However, the use of single LAB as starter culture showed varied results (Latief et al. 2023). The use of other LAB species such as *Lactobacillus rhamnosus* in coffee bean fermentation has been documented (de Carvalho Neto et al. 2018; Elhalis, Cox, and Zhao 2022). Wang et al. (2019) reported that the fermentation of Arabica GCB with *L. rhamnosus* HN001 resulted in increased acidity, attributed to elevated concentrations of organic acids relative to the control. Similarly, co‐fermentation with *L. mesenteroides*, *L. plantarum*, and *Pediococcus pentosaceus*, alongside yeasts such as *S. cerevisiae*, *Pichia guilliermondii*, and *Candida parapsilosis*, led to enhanced organic acid production in Arabica GCB. Notably, *Wickerhamomyces anomalus* and *Rhodotorula mucilaginosa* demonstrated compatibility with *Enterococcus mundtii*, further promoting acid formation. In contrast, fermentation with *Lactococcus lactis* subsp. *cremoris* ATCC 19257 yielded coffee beans with reduced acidity compared to the control (C. Wang et al. 2020b). These findings highlight the importance of considering both the initial organic acid content of the beans and the specific microbial consortia employed during fermentation to achieve targeted acidity levels. Notably, fermenting coffee beans with inherently high acidity may paradoxically yield a final product with reduced acidity, offering a potential strategy for producing low‐acid coffee suitable for acid‐sensitive consumers. Conversely, applying LAB‐based fermentation to Liberoid coffee beans may enhance their acidity and sensory complexity, potentially enabling the development of Arabica‐like flavor profiles and supporting Liberoid's viability as an alternative to Arabica coffee.

#### 6.2.3 Microorganisms Targeting Mouthfeel

Mouthfeel, as defined by the SCA (2024), refers to the tactile perception of the brew related to thickness (viscosity), texture, and mouth‐drying properties (astringency). Texture refers to the specific quality of mouthfeel, such as roughness (gritty, sandy), oiliness, and smoothness, while the intensity of these tactile sensations perceived during tasting is referred to as “body.” Hence, the occurrence of polysaccharides, lipids, phenolic compounds, and melanoidins is a determinant factor affecting the mouthfeel. Structural conformation and extractability of these components may be a more significant determinant of mouthfeel than their concentration. In their natural forms, polysaccharides such as galactomannans and arabinogalactans were reported to be responsible for viscosity and emulsifying properties of coffee (Lin et al. 2025). However, given that the concentrations of these compounds in GCB are comparable (Table 2), also in terms of structural conformation (Fischer et al. 2001), the factors determining the direct effect on mouthfeel are the roasting and brewing processes, which affect their structural changes and extractability. Furthermore, interactions of these specific‐tactile contributors could counteract each other; for example, oily‐contributing component such as linoleic acid (Várady et al. 2024) may offset the crisp and bright sensations contributed by organic acids, or vice versa (Echeverri‐Giraldo et al. 2024; Wei et al. 2025).

The application of microorganisms capable of degrading proteins and polysaccharides helped to release these components from cellulose/hemicellulose matrix, improving their dissolvability; these hold promises for enhancing the mouthfeel of coffee. Furthermore, fermentation may also facilitate the release of lipids from cellular matrix. Lipids are key contributors to coffee mouthfeel. When present at higher levels, they increase body and promote a smoother, more oily texture (Fabella‐Garcia et al. 2025). Extensive fermentation‐derived compounds such as amino acids and reducing sugars later serve as precursors for melanoidin formation via Maillard non‐enzymatic browning during roasting. Elevated melanoidin concentrations in RBs may increase total dissolved solids in the brew, thereby improving perceived mouthfeel. While the addition of microorganisms in coffee bean fermentation mostly assists the degradation of macromolecules by their production of polysaccharide and protein degrading enzymes, some microorganisms contribute to the coffee flavor through bioconversion toward secondary metabolites. For example, *Limosilactobacillus fermentum* has been reported to produce phenolic acids (Dorawa and Kolniak‐Ostek 2026), which are responsible for mouth‐drying properties in coffee. In Liberoid coffee, the fermentation of GCBs with *Alcaligenes* sp. and *Exiguobacterium indicum* enhanced mouthfeel, although the resulting brews exhibited reduced acidity (Tarigan, Adriliana, et al. 2024). This suggests a potential inverse relationship between acidity and mouthfeel, warranting further investigation. In contrast, a study employing *S. cerevisiae* as a starter culture in the fermentation of Arabica GCBs reported a significant reduction in mouthfeel, despite improvements in fruity and acidic sensory attributes (Moreno et al. 2025). These findings underscore the importance of microbial selection and matrix‐specific responses in modulating sensory outcomes. Further research is needed to elucidate the mechanistic links between microbial metabolism, organic acid dynamics, and mouthfeel development, particularly in the context of Liberoid coffee fermentation.

#### 6.2.4 Microorganisms Targeting Bitterness

Bitterness in coffee is primarily influenced by the concentration of bioactive compounds such as CGAs and methylxanthines, as well as the relative abundance of simple sugars. Liberoid coffee has been reported to exhibit pronounced bitterness and lingering aftertaste, which may be attributed to its elevated CGA content. Efforts to identify and reduce CGA‐related bitterness have been made (Ji et al. 2023; Linne et al. 2023). A method for CGA reduction based on esterase activity was found to be promising (Kraehenbuehl et al. 2017). Incorporating esterase‐producing microorganisms into in vitro fermentation protocols offers a promising strategy for mitigating this sensory attribute. They included *Lactobacillus reuteri* DSM 20016, *Bacteroides fragilis* ATCC 25285, and *Bifidobacterium longum* subsp. *infantis* DSM 20088 (Balaj et al. 2022). These microorganisms produce esterase (such as cinnamoyl esterase), an important enzyme to degrade the CGAs (Kraehenbuehl et al. 2017; Balaj et al. 2022). Despite its low concentration in Liberoid coffee, the occurrence of caffeine may still contribute to the bitterness. In this aspect, the use of starter culture has shown positive effects in reducing caffeine content in coffee beans. Notably, *Pseudomonas putida* and *Alcaligenes* spp. have demonstrated biodecaffeination capabilities via N‐demethylation and C‐8 oxidation pathways (Ran et al. 2025), enabling partial degradation of caffeine and related methylxanthines. This approach may reduce bitterness while preserving the overall sensory integrity of the beans, offering a viable alternative to conventional decaffeination methods, which often compromise flavor quality. Such innovations are particularly relevant for caffeine‐sensitive consumers seeking low‐bitter coffee options.

As discussed in Section 5.5.2.2, EPMs, particularly in vitro fermentation, promote the release of bound phenolic compounds and markedly increase TPC in GCB, a change generally associated with heightened bitterness and astringency. However, previous studies have reported variable outcomes (Wang et al. 2019; Calderon et al. 2023; Tarigan, Adriliana, et al. 2024; Moreno et al. 2025), likely due to differences in chemical composition, including sugar concentrations. Enhanced levels of simple sugars, resulting from microbial polysaccharide degradation, may mask bitterness, modulating the final sensory perception. These findings underscore the need for targeted microbial selection and compositional profiling to optimize bitterness in Liberoid coffee fermentation.

#### 6.2.5 Future Direction for the Development of Starter Cultures

In vitro fermentation presents a versatile approach for enhancing the sensory and compositional attributes of Liberoid coffee beans. However, several critical considerations must be addressed to ensure process safety and efficacy. The selection of starter cultures should prioritize microbial safety, supported by a comprehensive understanding of their metabolic pathways prior to application. Fermentation conditions must be tightly controlled to guide microbial activity along intended biochemical routes, as deviations may result in the formation of undesirable or harmful metabolites (Skowron et al. 2022). The use of mixed microbial cultures warrants careful evaluation, as interspecies interactions, whether synergistic or antagonistic, can significantly influence fermentation outcomes. Investigating alternative strategies for co‐culturing microorganisms may offer improved control over metabolic dynamics and enhance the reproducibility of desired flavor profiles in Liberoid coffee. Continued research into microbial compatibility and pathway regulation is essential to optimize in vitro fermentation for this underutilized coffee species.

The incorporation of commercially available microorganisms offers significant potential to enhance the efficacy of in vitro fermentation for developing novel Liberoid‐based coffee products with targeted sensory and functional attributes. The wine industry provides a robust precedent for microbial utilization, having established extensive databases of yeasts and lactic acid bacteria optimized for flavor modulation (H. Zhao et al. 2025). In this regard, various promising microorganisms have been evaluated as starter cultures for wine production, some of which may also offer significant benefits for coffee fermentation. Species such as *Candida glabrata*, *Hanseniaspora* spp., and *Zygosaccharomyces rouxii*, known for their capacity to elevate ester synthesis, may be particularly beneficial for improving the aromatic complexity of fermented coffee beans. The use of hybrid yeast strains also presents a promising avenue. Hybrids including *S. cerevisiae* × *Metschnikowia pulcherrima* (GY7), *S. cerevisiae* × *Hanseniaspora uvarum* (71B), *S. cerevisiae* × *Lachancea thermotolerans* (CY38), and *S. cerevisiae* × *Pichia kudriavzevii* (CY515) have demonstrated the ability to preserve and enhance phenolic and flavonoid contents (H. Zhao et al. 2025), supporting the development of coffee with improved health‐promoting properties.

The optimization of fermentation conditions is critical when applying selected microorganisms to Liberoid coffee beans, due to the distinct chemical composition and structural characteristics of this substrate. Tailored process parameters are necessary to ensure consistent microbial performance and to achieve targeted compositional and sensory outcomes. The integration of AI technology is needed to accelerate this effort (Motta et al. 2025; Pino and Apraez 2025). AI capabilities for data science, especially in big data analysis, are essential. This will be useful for determining the interaction of multifactorial datasets. Hence, deep analysis on microorganisms’ complex pathways in relation to chemical composition, structural changes, and sensory analysis can be done to support the optimization of Liberoid coffee processing for desired properties.

From an industrial perspective, the transformation of low‐quality or highly defective coffee beans into high value‐added products is particularly attractive. In vitro fermentation using well‐characterized microbial cultures offers a controlled approach to modulate flavor and reduce sensory defects, thereby enabling the utilization of lower grade raw materials for cost‐effective and sustainable production. Moreover, the broad diversity of available microorganisms presents extensive opportunities for product innovation. Strategic selection and application of microbial strains can support the development of differentiated Liberoid‐based coffee products, expanding consumer choices and enhancing market potential.

## 7 Other Limitations in the Liberoid Coffee Knowledge

Besides the gaps already described in previous sections, Liberoid coffee is considered a novel coffee commodity. Thus, detailed studies on its chemical composition are still limited; less is known for its sensory quality. Comprehensive studies on the genetic diversity of Liberoid coffee beans are urgently needed; comparative analysis between Liberica and Excelsa should be prioritized; further, the re‐classification of existing Liberica and Excelsa coffee varieties currently cultivated (e.g., in Indonesia) should be done following the current classification of *C. liberica* var *liberica*, *C. liberica* var. *dwevrei* and C. *liberica* var *klainei*. This will serve as important baseline for the optimization of Liberoid coffee beans in terms of processing and quality.

Limitations in the current cultivation and breeding practices of Liberoid coffee present significant challenges to its scientific characterization and sensory evaluation. On‐farm availability of Liberoid is typically low, and the genetic identity of planted material, whether genotype, clone, or variety, is often undocumented. This lack of traceability hinders the exclusive processing of Liberoid beans and leads to frequent mixing with Arabica and Robusta, with little attention to varietal sorting. Liberoid coffee is commonly intercropped with Robusta, resulting in frequent misclassification at the farm level. Historical taxonomic ambiguities within Liberoid have further complicated differentiation from Arabica and Robusta, as well as among Liberoid types themselves. These factors contribute to inconsistent and sometimes conflicting reports on sensory quality. Given the prevalence of unclear genetic identification in existing studies, caution is warranted when interpreting and generalizing sensory and compositional findings related to Liberoid coffee.

Differences in the analytical methods used for determining bioactive compounds limit comparative analysis among currently available studies. Variations in extraction methods, units (wet basis, DB, and free‐fat DB), sample preparations, and sample matrices (powder, extracts, infusion) can significantly interfere with the accurate quantification of some compounds. The quantification of bioactive and volatile compounds often lacks proper standardization (internal, external, and authentic standards), making the results obtained from similar studies difficult to compare. Furthermore, sample preparation methods should be selected cautiously and optimized specifically for the analysis of targeted bioactive compounds. This is necessary to avoid the possibility of altering chemical profiles due to temperature, prolonged extraction/analysis time, or the use of improper solvents/chemicals.

## 8 Conclusions

Liberoid coffee represents a promising yet underutilized resource in the global coffee market, offering unique sensory profiles and adaptability to marginal environments. This review highlights the critical role of genetic variation and post‐harvest processing in shaping its chemical and sensory properties. Conventional processing methods, often adapted from Arabica and Robusta practices, are suboptimal for Liberoid due to its distinct cherry morphology and pulp composition. Emerging approaches such as in vitro fermentation provide a flexible and targeted strategy to enhance flavor, reduce bitterness, and improve mouthfeel, particularly when supported by microbial selection and process optimization.

However, several challenges must be addressed to fully realize Liberoid coffee's potential. These include the lack of standardized genetic identification, limited compositional data, and insufficient control over microbial fermentation pathways. Future research should prioritize the development of genotype‐specific processing protocols, microbial safety assessments, and fermentation models tailored to Liberoid substrates. The utilization of AI is prospective; its capabilities in processing multifactorial big data are essential in accelerating the optimization of processing, the prediction of quality, and the determination of suitable microorganisms and pathways to obtain a product with desired properties. Integrating these strategies will support the production of high‐quality, differentiated Liberoid coffee products, contributing to sustainable value creation and diversification in the coffee industry.

## Author Contributions

**Noor Ariefandie Febrianto**: conceptualization, methodology, writing – original draft, writing – review and editing, visualization, data curation. **Fan Zhu**: conceptualization, writing – review and editing, supervision.

## Conflicts of Interest

The authors declare no conflicts of interest.

## Funding

The authors have nothing to report.

## Supporting information




**Supplementary Figure**: crf370503‐sup‐0001‐FigureS1.pdf
