# Supplementary material for: Unlocking the Quality Potential of Liberoid Coffee: Advances in Composition, Processing, and Microbial Fermentation
Source: Compr Rev Food Sci Food Saf. 2026 May 13;25:e70503. doi: 10.1111/1541-4337.70503 (PMC13172441; doi:10.1111/1541-4337.70503)
Supplement: Supplementary file 1 — Supplementary Figure: crf370503‐sup‐0001‐FigureS1.pdf [file CRF3-25-e70503-s001.pdf]

Supplementary Material

Unlocking the quality potential of Liberoid coffee: Advances in composition, processing, and microbial fermentation

Noor Ariefandie Febrianto[1], Fan Zhu[2*]

[1]Indonesian Coffee and Cocoa Research Institute (ICCRI), PT. Riset Perkebunan Nusantara, Jember, East Java, Indonesia

[2]School of Chemical Sciences, The University of Auckland, Private Bag 92019, Auckland 1142, New Zealand

*Email: fzhu5@yahoo.com

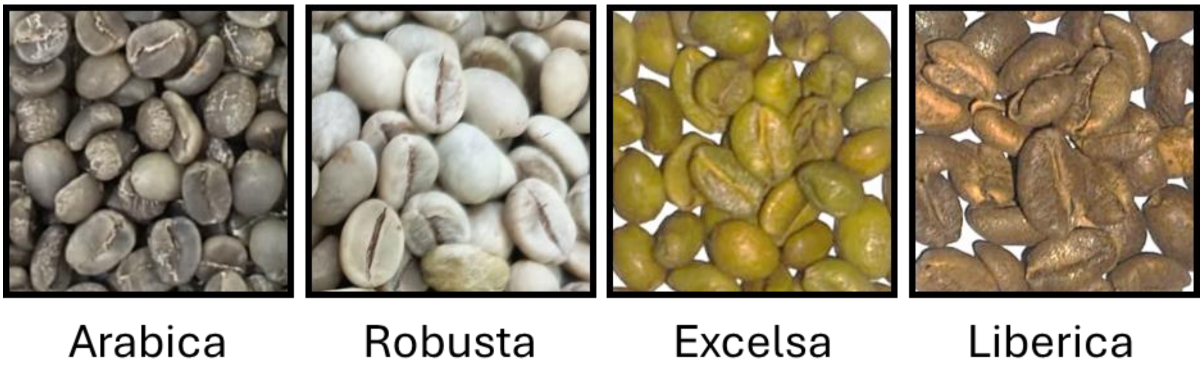

Supplementary Figure 1. The comparison of physical appearance of green coffee beans obtained from different varieties. The images are obtained as the properties of Indonesian Coffee and Cocoa Research Institute.
